# Integrative In Silico Analysis to Identify Functional and Structural Impacts of nsSNPs on Programmed Cell Death Protein 1 (PD-1) Protein and UTRs: Potential Biomarkers for Cancer Susceptibility

**DOI:** 10.3390/genes16030307

**Published:** 2025-03-04

**Authors:** Hakeemah Al-Nakhle, Retaj Al-Shahrani, Jawanah Al-Ahmadi, Wesal Al-Madani, Rufayda Al-Juhani

**Affiliations:** Department of Clinical Laboratory Sciences, College of Applied Medical Sciences, Taibah University, Al-Madinah Al-Monawarah 42353, Saudi Arabia

**Keywords:** programmed cell death protein 1, nsSNPs, immune checkpoint regulation, cancer susceptibility, biomarkers

## Abstract

**Background:** Programmed cell death protein 1 (PD-1), encoded by the *PDCD1* gene, is critical in immune checkpoint regulation and cancer immune evasion. Variants in *PDCD1* may alter its function, impacting cancer susceptibility and disease progression. **Objectives:** This study evaluates the structural, functional, and regulatory impacts of non-synonymous single-nucleotide polymorphisms (nsSNPs) in the *PDCD1* gene, focusing on their pathogenic and oncogenic roles. **Methods:** Computational tools, including PredictSNP1.0, I-Mutant2.0, MUpro, HOPE, MutPred2, Cscape, Cscape-Somatic, GEPIA2, cBioPortal, and STRING, were used to analyze 695 nsSNPs in the PD1 protein. The analysis covered structural impacts, stability changes, regulatory effects, and oncogenic potential, focusing on conserved domains and protein–ligand interactions. **Results:** The analysis identified 84 deleterious variants, with 45 mapped to conserved regions like the Ig V-set domain essential for ligand-binding interactions. Stability analyses identified 78 destabilizing variants with significant protein instability (ΔΔG values). Ten nsSNPs were identified as potential cancer drivers. Expression profiling showed differential *PDCD1* expression in tumor versus normal tissues, correlating with improved survival in skin melanoma but limited value in ovarian cancer. Regulatory SNPs disrupted miRNA-binding sites and transcriptional regulation, affecting *PDCD1* expression. STRING analysis revealed key PD-1 protein partners within immune pathways, including PD-L1 and PD-L2. **Conclusions:** This study highlights the significance of *PDCD1* nsSNPs as potential biomarkers for cancer susceptibility, advancing the understanding of PD-1 regulation. Experimental validation and multi-omics integration are crucial to refine these findings and enhance theraputic strategies.

## 1. Introduction

Programmed cell death protein 1 (PD-1), encoded by the *PDCD1* gene, which is also known as *CD279*, is a key member of the CD28/B7 superfamily of costimulatory molecules that play a critical role in T-cell regulation [1]. PD-1 is expressed on different immune cells, such as activated CD4+ T cells, CD8+ T cells, natural killer T (NKT) cells, B cells, and activated monocytes [2,3]. The human *PDCD1* gene is located on chromosome 2q37.3 and encodes a 55 kDa type I transmembrane receptor protein consisting of 288 amino acids [4,5].

Structurally, PD-1 has an extracellular domain that contains an IgV-like region and a cytoplasmic domain that has two key motifs, namely, the immunoreceptor tyrosine-based inhibitory motif (ITIM) and the immunoreceptor tyrosine-based switch motif (ITSM) [1,6]. Additionally, PD-1 interacts with its ligands, programmed death-ligand 1 (PD-L1) (B7-H1; *CD274*) and programmed death-ligand 2 (PD-L2) (B7-DC; *CD273*), which are expressed on antigen-presenting cells (APCs) and, in some cases, tumor cells to mediate immunosuppression [7,8]. This interaction activates the cytoplasmic ITIM, stimulating inhibitory signals that reduce T-lymphocyte activation and proliferation, inhibit cytokine production, and enhance T-cell apoptosis. These mechanisms collectively contribute to maintaining peripheral tolerance [8].

PD-L1 is frequently upregulated by tumor cells as an adaptive mechanism to evade immune surveillance while increasing PD-1 expression on immune cells, leading to CD8+ T-cell exhaustion, impairing antitumor immune responses, and stimulating tumor progression [9]. The binding of PD-L1 on tumor cells to PD-1 on tumor-infiltrating T cells (TILs) promotes the activation of Src homology region 2 domain-containing phosphatases (SHP2), which inhibits the T-cell receptor (TCR) signaling pathway and inhibits the function of T cells. This disruption of immune surveillance through PD-L1/PD-1 signaling allows for tumor progression and enhances cancer cells’ survival [10].

nsSNPs are genetic alterations that lead to a change in the amino acid sequence of a protein [11]. These changes can impact the protein’s structure, stability, and function, changing biological processes [11]. They can impact the regulation of gene expression at the transcriptional level, and several studies have established the relationship between polymorphisms in the *PDCD1* and *CD274* genes and the risk of developing different types of cancer and other immune-related diseases [12,13,14,15]. Additionally, nsSNPs in the human *PDCD1* gene may affect its functions, especially immune regulation, cancer susceptibility, and therapy response. In the context of the PD-1 protein, nsSNPs can affect its role in immune checkpoint regulation, potentially impacting the effectiveness of cancer immunotherapies or ligand binding. A limited number of polymorphisms have been investigated for their associations with cancer and immune-related diseases. Notably, the PD-1.5 (rs2227981), PD-1.6 (rs10204525), and PD-1.9 (rs2227982) polymorphisms are associated with several cancers, including lung adenocarcinoma [16], cervical cancer [17], gastric cancer [18], and thyroid cancer [19].

A meta-analysis study examined the correlation between PD-1 gene polymorphisms and cancer susceptibility by analyzing case–control studies. The analysis revealed that the PD-1 rs2227981 and rs11568821 were associated with a decreased overall risk of cancer, while the PD-1 rs7421861 was associated with an increased susceptibility to cancer. However, no significant correlations were observed for the PD-1 rs2227982, rs36084323, or rs10204525 polymorphisms [14].

The upregulation of PD-1 has been proposed to be a potential biomarker for acute myeloid leukemia (AML), as the PD-1 expression was upregulated in patients as compared to healthy controls. Furthermore, the rs36084323 polymorphism has been linked with an increased risk of AML [20]. In addition, the PD-1 rs2227982 has been found to have a significant association with tumor size, while the rs2227981 and rs11568821 polymorphisms are related to tumor grade and stage [21]. In ovarian cancer, the polymorphisms PD-1 rs2227982 and PD-L1 rs4143815 have been associated with increased cancer susceptibility and poor prognosis, particularly when PD-1 and PD-L1 expression levels are reduced [22]. Conversely, high PD-1/PD-L1 expression is associated with favorable outcomes in ovarian cancer [22].

SNPs are distributed across various regions of genes, including promoters, exons, and introns, as well as the 5′ and 3′ untranslated regions (UTRs). Their effects on gene expression and cancer susceptibility vary based on their specific position. Promoter-region SNPs can regulate gene expression by altering promoter activity, affecting transcription factor binding, DNA methylation patterns, and histone modifications [23,24,25,26]. Missense SNPs located in exonic regions contribute to cancer susceptibility by affecting gene transcription and translation, leading to alterations in protein expression and function. SNPs in the 5′UTR impact translation efficiency, while those in the 3′UTR affect the binding of miRNAs to target genes, thereby regulating gene expression [27]. SNPs in the *PDCD-1* gene significantly influence its protein expression and function, affecting immune regulation and cancer progression. For instance, the rs36084323 (−606 A > G) in the promoter region increases transcription by altering the binding of transcription factors, thereby leading to the upregulation of PD-1 expression [28]. This contributes to cancer immune evasion and has been found to be associated with poor survival outcomes and increased recurrence rates in cancers such as hepatocellular carcinoma (HCC) [29] and non-small-cell lung cancer (NSCLC), including squamous-cell carcinoma (SCC) [30].

Several SNPs have been identified in miRNA-binding sites within the 3′ UTR of the PD-L1 gene, including rs2297136 and rs4742098. These SNPs affect the binding of miRNAs, particularly miR-296-5p and miR-138, thereby influencing PD-L1 expression, increasing the risk of developing NSCLC, metastasis, and poor prognosis [31]. Similarly, miR-4717 has been shown to regulate PD-1 expression through interactions with the 3′ UTR, modulating the immune responses and disease progression in cases of chronic hepatitis B virus (HBV) infection [32]. It has also been suggested that increased PD-1 expression may play a role in the progression of chronic HBV infection by enhancing viral replication. This process has been associated with polymorphisms in the PD-1 3′ UTR [33]. A recent study also revealed that Mediterranean HBV-infected subjects with PD-1 GG and GA genotypes at rs10204525 exhibited elevated PD-1 mRNA expression, which may increase their susceptibility to developing chronic HBV infection [34].

Post-translational modifications (PTMs), particularly glycosylation, are important in PD-1’s function, including its localization, stability, and ligand interactions [35]. Glycosylation plays an essential role in regulating glycoproteins, particularly in immune checkpoint receptors such as PD-1. Dysregulation of glycosylation contributes to cancer progression through the modulation of ligand–receptor binding, and by impairing T-cell function [36,37].

The binding of PD-1 to its ligands is regulated by glycosylation at the N49, N58, N74, and N116 sites in the PD-1 IgV domain [38]. More specifically, core fucosylation at N49 and N74 regulates PD-1 expression. Targeted inhibition of this core-fucosylation using 2-fluoro-L-fucose (2F-Fuc), a specific inhibitor of fucosyltransferase FUT8, has been shown to downregulate PD-1 expression and increase the activation of T cells [39]. Interestingly, glycosylation affects the binding affinity of cemiplimab, an anti-PD-1 antibody at the N58 glycosylation site, underscoring its therapeutic significance [40]. However, the diversity of glycans complicates glycosylation-targeted therapies.

Phosphorylation of the tyrosine within the ITSM domain of PD-1 at Y248 is essential for immunosuppressive signaling [41]. Upon PD-1’s binding to PD-L1, the ITSM recruits SHP2 to the binding site, which results in the dephosphorylation of proteins such as ZAP70 and CD28, consequently inhibiting IL-2 production, glucose metabolism, and T-cell function [42,43]. SHP1 can compensate for SHP2’s absence, maintaining PD-1’s role in immune inhibition [44].

The impact of PD-1 nsSNPs on PTMs is not well understood; therefore, it is not possible to determine how these variations might affect PD-1’s function and the overall outcome of immunotherapy. Additionally, nsSNPs in PD-1 can affect glycosylation sites that are important for proteins’ stability, localization, and interaction with ligands (PD-L1/PD-L2) [45]. These alterations in glycosylation result in the dysregulation of PD-1’s immunoregulatory function and, therefore, alter the efficacy of PD-1-targeted therapies such as monoclonal antibodies. Due to the vital role of PD-1 in the modulation of immune regulation, it has become a significant therapeutic target in cancer immunotherapy. Several checkpoint inhibitors, such as nivolumab and pembrolizumab, have achieved significant success in reactivating T cells to target cancer cells [9].

nsSNPs can be classified as passengers or drivers based on their functional role, especially in cases of cancer development [46]. For instance, driver SNPs have a direct functional effect that is responsible for disease progression [46,47]. They often affect the protein’s function or expression, which, in turn, results in changes in phenotype, such as activating oncogenes and inactivating tumor-suppressor genes in cancer. In contrast, passenger SNPs have no direct effect on the biological function or disease; they occur randomly and are carried along with driver mutations [47,48,49].

Although significant advances have been made in understanding the role of PD-1 in the regulation of immunity and cancer immunotherapy, there are still many unanswered questions concerning how specific nsSNPs affect PD-1’s protein stability, function, and structure. These gaps hinder the full exploitation of PD-1 as a therapeutic target and the development of more effective and personalized immunotherapies. Even though numerous nsSNPs in the *PDCD1* gene have been identified, the majority of these variants have not been extensively characterized in terms of their pathogenicity and their specific effects on PD-1’s protein function, structure, and stability. Many studies focus on a few well-known polymorphisms, leaving a vast number of potentially impactful nsSNPs underexplored.

Addressing these gaps requires computational methods for modeling the consequences of nsSNPs on proteins’ structure, function, and stability. These methods help the researchers to know how genetic variations can affect proteins’ stability, interactions, and overall functionality, with a focus on diseases such as cancer.

The primary objective of this study was to determine the effect of nsSNPs on the structure, stability, and function of the PD-1 protein, with emphasis on their pathogenic and oncogenic implications. Furthermore, this study aimed to evaluate the structural alterations induced by nsSNPs and their effects on the conformational dynamics and stability of the PD-1 protein. This study further explored the oncogenic potential of the examined nsSNPs, identifying their possible associations with various cancer types. Additionally, this study examined the expression profiles of *PDCD1* across cancer types and their association with overall survival rates to examine the prognostic value of PD-1. In addition to the coding regions, the analysis involved the effects of 3’UTR variants on microRNA-binding sites and the identification of functional nsSNPs in non-coding regions, which may regulate gene expression. Finally, the molecular pathogenicity of these nsSNPs was assessed to understand their contribution to disease mechanisms. Collectively, this study offers a comprehensive analysis of how nsSNPs influence the PD-1 protein, thereby enhancing our understanding of cancer development and guiding future therapeutic innovations.

## 2. Materials and Methods

### 2.1. Retrieval of PD-1 nsSNPs from the Database

**Figure 1:**
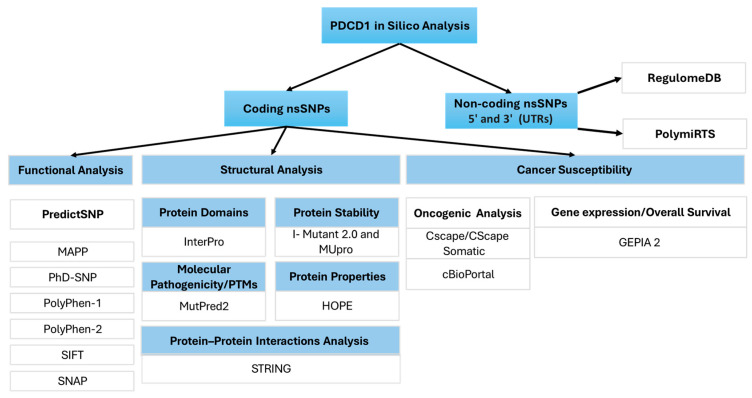
A workflow representing all of the in silico tools utilized in this study.

The dataset of nsSNPs for the *PDCD1* gene was extracted from the ENSEMBL database, using the accession number ENSG00000188389 [50]. To select the relevant missense nsSNPs, a missense filter was used to narrow down the dataset. The corresponding protein sequence for the *PDCD1* gene was retrieved in FASTA format from the UniProt database, using the ID Q15116. To analyze non-coding SNPs, other data were also collected from the ENSEMBL database, and the SNPs were filtered to include only SNPs in the 5′ and 3′ UTRs and with a minor allelic frequency (MAF) of ≤0.001. The GnomAD-Exomes database (gnomAD v4.1) was utilized to obtain information on the global frequencies of the examined PD-1 nsSNPs. A detailed overview of the methodology is presented in Figure 1.

### 2.2. Determining the Most Deleterious SNPs

To assess whether the identified nsSNPs were likely deleterious or benign, the PredictSNP1.0 tools (https://loschmidt.chemi.muni.cz/predictsnp/, accessed on 25 January 2024) [51] were used. PredictSNP1.0 is a consensus SNP classifier that integrates predictions from six well-established computational tools, including SIFT, PolyPhen-1, PolyPhen-2, MAPP, PhD-SNP, and SNAP. Thus, PredictSNP1.0 integrates the predictions of these individual prediction models, which increases the accuracy of the deleteriousness predictions and provides a reliable approach to identify the nsSNPs that are most likely to affect the protein function significantly. A consensus-based approach combines the unique strengths of various tools that analyze factors such as the conservation of residues, structural consequences, and the effects on stability, enabling more precise identification of high-risk nsSNPs.

### 2.3. The Identification of nsSNPs Within the Domains of the PD-1 Protein

To identify nsSNPs that are located in the conserved domains of the PD-1 protein, the InterPro tool [52] (https://www.ebi.ac.uk/interpro/, accessed on 1 February 2024) was used. InterPro is an integrated database that comprises several member databases, such as the immunoglobulin-like domain, immunoglobulin V-set domain, and immunoglobulin subtype, to identify the protein motifs and domains. This integration enhances the functional annotation of the protein regions. In this study, the PD-1 protein sequence was input in the FASTA format, enabling the accurate positioning of nsSNPs in the conserved functional domains.

### 2.4. Analyzing the Effects of the nsSNPs on PD-1 Protein Stability

To assess the effects of deleterious nsSNPs on the stability of the PD-1 protein, we employed two computational tools: I-Mutant 2.0 (https://folding.biofold.org/i-mutant/i-mutant2.0.html) [53] and Mupro1.1 (http://mupro.proteomics.ics.uci.edu/), (both accessed on 9 February 2024) [54]. I-Mutant 2.0 is a support vector machine (SVM)-based server that estimates the change in free energy (ΔΔG) to predict stability changes. A ΔΔG value less than 0 (kcal/mol) means decreased protein stability, while a value greater than 0 signifies increased stability. The analysis was conducted at 25 °C and pH 7.0, and the results were classified based on ΔΔG values, binary stability predictions, and a reliability index (RI) for each result, thus determining the deleterious nsSNPs that lead to instability of the protein.

Additionally, we utilized MUpro, a server that predicts the effects of mutations on protein stability without requiring the protein’s tertiary structure. This tool uses SVM and neural network classifiers that were trained on large datasets of mutations, and it has an accuracy rate of over 84%. MUpro estimates stability changes by the ΔΔG value and gives a confidence score between −1 and 1. Scores below 0 suggest a destabilizing effect, while scores above 0 indicate increased stability. All of these tools collectively enabled a comprehensive assessment of how nsSNP-induced mutations potentially alter PD-1 protein stability.

### 2.5. Investigating the Impact of nsSNPs on the 3D Structure of the PD-1 Protein

To determine the structural impact of nsSNPs on the PD-1 protein, we utilized the HOPE software (Have Our Protein Explained) (https://www3.cmbi.umcn.nl/hope/, accessed on 15 February 2024) [55]. HOPE is a web-based tool that analyzes how specific mutations may alter protein structure. The analysis requires the input of the protein sequence in FASTA format, with detailed SNP information. HOPE integrates data from multiple sources, including UniProt and the Distributed Annotation System (DAS), and uses predictive algorithms to produce a 3D visualization of the mutated protein structure. This allows for a comprehensive assessment of the structural changes induced by nsSNPs.

### 2.6. Evaluation of Molecular Pathogenicity of nsSNPs

To understand the molecular consequences of amino acid substitutions in the PD-1 protein, we used MutPred2 (http://mutpred.mutdb.org/index.html, accessed on 22 February 2024) [56,57]. This tool determines whether certain amino acid changes may result in the gain or loss of particular phenotypic features in proteins, applying a significance threshold of *p* < 0.05. MutPred2 evaluates various structural and functional characteristics, including the secondary structure, the transmembrane regions, signal peptides, catalytic sites, binding affinities (for both macromolecules and metals), PTMs, and allosteric effects. Amino acid substitutions can lead to various effects on protein function, including alterations in protein conformation, disruption of molecular interactions, or loss of PTM sites, potentially impacting protein function at the phenotypic level.

For our analysis, we submitted the human PD-1 protein sequence in the FASTA format and the information about certain single amino acid substitutions in MutPred2. The tool calculated a probability score that each substitution is damaging or disease-related, along with a list of possible molecular impacts ranked by *p*-value, all significantly below the 0.05 threshold.

### 2.7. Oncogenic and Phenotypic Analysis

To determine the oncogenic potential of the identified nsSNPs, we used Cscape, available at (http://cscape.biocompute.org.uk/) [58], and Cscape-Somatic, available at (http://cscape-somatic.biocompute.org.uk) [59] (both accessed on 3 March 2024). CScape assigns scores to individual nsSNPs, classifying them as high-confidence oncogenic, benign, or malignant, with an approximate accuracy of 92% for coding regions and 76% for non-coding regions. A higher score means a higher oncogenic potential. In contrast, Cscape-Somatic can specifically predict whether a certain point mutation is oncogenic (disease-causing) or neutral, providing more specific information about the pathogenicity of these variants.

Cscape uses data in the format of chromosome, position, reference, and mutation, using the GRCh38 assembly of the genome. Predictions are given as *p*-values between 0 and 1, where *p*-values greater than 0.5 are classified as disease-causing, while those less than 0.5 are considered benign. However, Cscape-Somatic can identify cancer-driving mutations from the pool of somatic and passenger mutations, which are more often observed in the later stages of the tumor, with less carcinogenic potential. Even though it works similarly to Cscape, Cscape-Somatic employs GRCh37 assembly and outputs *p*-scores between 0 and 1, where the scores >0.5 and <0.5 are classified as oncogenic and passenger mutations, respectively.

### 2.8. Identification of Cancer and Association with nsSNPs

The cBioPortal database (https://www.cbioportal.org/, accessed on 5 November 2024) [60,61] is available to the public and provides facilities for retrieving, viewing, and processing big data in cancer genomics. In this study, we examined the distribution of *PDCD1* gene mutations or nsSNPs using the lollipop plot for mutation frequency available on the platform, aiming to identify potential associations between specific nsSNPs and cancer.

### 2.9. In Silico Analysis of PDCD1Gene Expression Profiles and Their Correlation with Survival Prognosis

To assess the prognostic value of *PDCD1* in cancer patients, gene expression profiles and patient survival data were analyzed using the Gene Expression Profiling Interactive Analysis (GEPIA2) website [62], which is based on the TCGA transcription database. This study examined the impact of *PDCD1* gene expression levels (high vs. low) on the overall survival rates of ovarian cancer (OV) and skin cutaneous melanoma (SKCM) patients, utilizing the Kaplan–Meier plot tool for analysis. This tool facilitates the visualization of gene expression profiles via dot plots or boxplots and supports survival analysis using the log-rank test. In this study, we input the candidate gene, PD-1, and selected specific cancer types (e.g., SKCM, OV) to perform both expression profiling and survival analysis.

### 2.10. Protein–Protein Interactions Analysis Using the Search Tool for the Retrieval of Interacting Genes/Proteins (STRING)

Protein–protein interactions are essential for the precise regulation of proteins’ biological functions. The STRING database (available at http://string-db.org, accessed on 1 March 2024) assists in analyzing protein interactions and creating networks of interacting proteins. The STRING database currently contains data on 24,584,628 proteins across 5090 species. In this research, we analyzed the PD-1 protein by uploading its FASTA sequence to STRING, which generated predicted interaction partners and assigned each a confidence score [63].

### 2.11. Functional and Pathway Enrichment Analysis Using STRING

The functional analysis focused on annotating and enriching proteins within the network based on their functional attributes. Enrichment was primarily performed using Gene Ontology (GO) terms, which encompass biological processes, molecular functions, and cellular components, as well as pathway-based analyses. To understand the physical and biological roles of the network, functional analysis is essential. Specifically, Gene Ontology (GO) and Kyoto Encyclopedia of Genes and Genomes (KEGG) pathway enrichment analyses were carried out using STRING.

### 2.12. Analysis of the Functional Relevance of Non-Coding SNPs (ncSNPs) in the PDCD1 Gene Regulatory Function Analysis Through RegulomeDB

We retrieved ncSNPs of the non-coding regions of the PDCD1 gene using data from the ENSEMBL database. The ncSNPs were then mapped to the regulatory elements of the human genome using RegulomeDB [64]. The input was SNP IDs with an MAF < 0.001 from 5′ or 3′ UTRs obtained from the Ensemble database. The output contained information such as chromosome position, dbSNP ID, rank, and score. It is crucial to identify functional variants in these regions, because such variants may alter the regulatory functions. RegulomeDB enables us to predict and rank the ncSNPs as regulatory elements by synthesizing data from multiple databases, including ENCODE ChIP-seq, FAIRE, DNase I hypersensitive sites, eQTLs, and dsQTLs. This ranking system classifies the ncSNPs into six categories depending on their potential impact on transcription factor binding or gene expression (Table S3).

### 2.13. Analysis of the Effect of 3UTR SNPs on microRNA (miRNA)-Binding Sites

To determine the effects of UTR variants on microRNA-binding sites, we used PolymiRTS Database 3.0 to analyze 3′ and 5′ UTR variants [65] (https://compbio.uthsc.edu/miRSNP/ accessed on 22 November 2024). The PolymiRTS tool requires a variant ID as an input, and the output generates details such as miRNA ID, functional classification, and the Context+ score. The consequences of these variants are classified into four functional classes, namely, “D”, “N”, “C”, and “O”. In particular, the “D” class means that the derived allele disrupts a conserved miRNA-binding site, the “N” class indicates disruption of a non-conserved site, the “C” class means that a new site for microRNA is generated, and the “O” class provides no ancestral allele information. Additionally, PolymiRTS offers a Context+ score, where more negative values indicate a higher likelihood of disease risk due to altered miRNA binding.

## 3. Results

### 3.1. Prediction of Functionally Important nsSNPs in the PDCD1 Gene

PredictSNP was utilized to evaluate 695 nsSNPs. Based on the consensus of all of the tools used, 84 nsSNPs were identified as deleterious, which strongly suggests that these variants are likely to affect the biological function of the PD-1 protein. Table 1 highlights these high-risk nsSNPs based on consensus predictions, emphasizing those potentially disrupting protein functions. Table S1 provides details on the proportion of high-risk nsSNPs identified by each tool, offering a comprehensive breakdown of each tool’s contributions to the deleterious classifications. Furthermore, Table S4 reports the allele frequency of seven nsSNPs in the coding region of *PDCD1* across genetic ancestry groups and sexes.

### 3.2. Identification of Structural and Functional Domains of the PD-1 Protein Using InterPro

**Figure 2:**
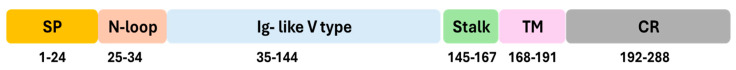
The schematic diagram illustrates the domain architecture of the PD-1 protein. The protein consists of the following regions: SP (signal peptide, orange), N-loop, Ig-like V type domain (blue), stalk (green), TM (transmembrane domain, pink), and CR (cytoplasmic region, gray).

Structural and functional analysis of the PD-1 protein domain was conducted through the InterPro tool. Four functionally important domains were identified: the non-cytoplasmic domain (1–167 residues) containing the Ig V-PD1 domain (35–144 residues), which facilitates ligand binding and immune checkpoint signaling by interacting with PD-L1 and PD-L2; the transmembrane region (TM)(168–191 residues), which anchors the protein in the cell membrane and ensures linkage between the outer and inner parts of the cell; and the cytosolic domain (192–288 residues), which receives the signal towards inhibition and, therefore, modifies the immune response accordingly. Conserved amino acids in these domains also highlight that PD-1 has to be structurally and functionally stable, as these regions are crucial in PD-1’s evolutionary development (Figure 2 and Figure S1).

### 3.3. Prediction of Impact of nsSNPs on PD-1 Protein Stability

Table 2 presents a detailed analysis of 78 missense nsSNPs in the PD-1 protein using the I-Mutant and MUpro computational tools. The information includes the SNP ID, the amino acid change, and the prediction of the stability of the protein. The tools assess whether the variants are destabilizing or stabilizing and provide ΔΔG (free energy change) values; negative ΔΔG indicates destabilization and positive ΔΔG indicates stabilization. Furthermore, the I-Mutant tool assigns a reliability index (RI), which is a confidence score from 0 to 10 for each prediction, with higher values indicating higher reliability.

The analysis reveals that the great majority of the examined nsSNPs are predicted to decrease PD-1 protein stability, as indicated by negative ΔΔG values presented for both tools (Table 2). The most destabilizing variants are W186G, with ΔΔG of −2.7 kcal/mol (I-Mutant) and −1.54 kcal/mol (MUpro), and F228S, with even more severe destabilization (ΔΔG of −2.99 kcal/mol (I-Mutant) and −1.32 kcal/mol (MUpro)). The most destabilizing mutations are also identified as L226Q and I251T, with both tools agreeing on their significant impact on protein stability.

Although most variants display decreasing PD-1 protein stability, certain variants exhibit minimal destabilization or even mild stabilization effects. For instance, N58I is predicted by the I-Mutant to increase stability, with a positive ΔΔG value of 1.25 kcal/mol, although MUpro predicts a slight decrease in stability. Some of the nsSNPs are context-dependent, highlighting the importance of using predictions from multiple tools in the evaluation process.

The strong agreement between I-Mutant and MUpro for most variants, combined with high reliability indices, supports the accuracy of these predictions. This analysis identifies key nsSNPs that may critically affect the stability of the PD-1 protein.

### 3.4. Structural Impacts of nsSNPs on PD-1 Protein According to Project HOPE

Through Project HOPE, we analyzed the structural impact of 78 nsSNPs in the PD-1 protein. Alterations in residue size, charge, hydrophobicity, and protein interactions were investigated, along with the conservation of the residues, to predict the likely structural and functional impact of these mutations. Our results show that 45 of the nsSNP residues are conserved. The detailed results of Project HOPE are presented in Table S3.

Mutations frequently resulted in significant changes in residue size and molecular interactions. For instance, nsSNPs such as L17P led to a loss of interactions due to smaller mutant residues, while larger residues introduced structural disruptions in cases like C54R. Hydrophobicity changes were also noticed, especially in conserved regions such as C54R, L42H, L65Q, and W286G, which had disrupted hydrogen bonding and poor folding efficiency. These nsSNPs were particularly damaging in conserved regions, disrupting key structural and functional features.

Residue-specific impacts on folding and core stability were prominent. Folding defects and destabilization resulted from incompatible spatial arrangements and the loss of hydrophobic interactions upon introducing larger residues into the protein core in nsSNPs such as C54R and L65Q. nsSNPs located in highly conserved residues, such as C54R, Y223H, and L226P, led to severe structural disruptions, whereas less conserved residues, such as D26G and N49H, primarily impacted localized protein regions without compromising overall stability. 

### 3.5. Predicting the Molecular Mechanisms of PD-1 nsSNP Pathogenicity Using MutPred2

The molecular effects of 45 nsSNPs on the PD-1 protein were analyzed using MutPred2, indicating significant alterations in molecular mechanisms in 17 nsSNPs with *p*-values < 0.05 (Table 3). Several key molecular mechanisms were determined, including alterations in transmembrane protein regions, with variants such as C54R disrupting membrane-spanning domains that are essential for PD-1’s function (e.g., C54R, *p* = 5.60 × 10^−4^; W67C, *p* = 1.20 × 10^−4^). Additionally, intrinsic disorder modifications, characterized by enhanced protein flexibility, were observed in variants such as Y68D (*p* = 7.90 × 10^−3^). Several nsSNPs caused the loss of structural elements, such as disulfide linkages at C54 (*p* = 5.50 × 10^−4^) and strand and loop disruptions in variants like W67C, leading to compromised protein stability.

**Figure 3:**
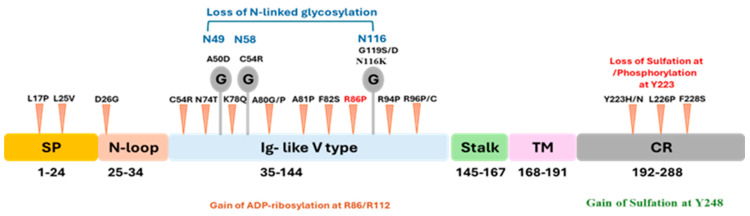
The PD-1 protein structure’s schematic representation illustrates key domains, post-translational modifications, and nsSNPs. Loss of N-linked glycosylation is indicated at residues N49, N58, and N116 (blue text). The nsSNPs associated with cancer driver mutations are shown as red arrows, highlighting critical residues affected by these variants. Gain of ADP-ribosylation is observed at residues R86/R112 (orange text). PTMs include loss of sulfation or phosphorylation at residue Y223 (red text) and gain of sulfation at residue Y248 (green text). Glycosylation sites, which play an essential role in PD-1 folding and ligand interaction, are marked by “G” symbols.

Several alterations in PTMs were also identified. Several nsSNPs demonstrated alterations in phosphorylation, glycosylation, GPI-anchor amidation, ADP-ribosylation, and sulfation sites as shown in Figure 3, including the loss of N-linked glycosylation at N58 (C54R, *p* = 5.6 × 10^−3^), gain of N-linked glycosylation at N116 (D117V, *p* = 0.02), loss of phosphorylation at Y223 (Y223N, *p* = 0.03), and loss of sulfation at Y223 (Y223S, *p* = 3.60 × 10^−3^). Gains in functional sites, including catalytic and binding sites, were observed in C54R (*p* = 0.02), which showed GPI-anchor amidation at N49 (*p* = 0.01).

High-impact variants such as C54R, Y68D, and W67C exhibited significant pathogenicity. For instance, C54R (MutPred2 Score: 0.962) demonstrated multiple disruptions, including loss of a disulfide linkage, altered transmembrane properties, and gain of a catalytic site. Y68D (MutPred2 Score: 0.925) exhibited intrinsic disorder gain and alterations in transmembrane and strand regions, while W67C (MutPred2 Score: 0.844) showed structural losses and gain of a disulfide linkage.

### 3.6. Oncogenic Potential of nsSNPs Using Cscape and Cscape- Somatic

Cscape and Cscape-Somatic analyses were conducted to determine the oncogenic potential of nsSNPs within the PD-1 protein. Using these tools, oncogenic nsSNPs were predicted based on coding scores, with a threshold of *p* > 0.5 defining harmful variants, and scores below this threshold indicating benign variants. The results of these analyses are presented in Table 4.

According to the CScape Somatic tool, many of the oncogenic variants identified in the analysis are driver mutations that actively contribute to tumorigenesis. For instance, L17P (CScape: 0.6021; driver: 0.839552), L25V (CScape: 0.581048; driver: 0.661509), C54R (CScape: 0.627091; driver: 0.50485), and A80P (CScape: 0.7654; driver: 0.679769), and L226P (Cscape: 0.80086; Driver: 0.631527) displayed exceptional oncogenic scores. However, some nsSNPs were classified as passenger mutations, indicating that they may not directly contribute to tumorigenesis despite their moderate oncogenic scores. Among these, L65P (CScape: 0.561139; passenger: 0.407533) and W67C (CScape: 0.68252; passenger: 0.341358) exhibited these characteristics. Several mutations appeared to be clear oncogenic drivers, including F228S (CScape: 0.730018; driver: 0.656042) and C241W (CScape: 0.662798; driver: 0.823574).

### 3.7. Association of the Damaging nsSNPs with Cancer

Using the cBioPortal web server, cancer-specific nsSNPs associated with the *PDCD1* gene were identified. Two nsSNPs, G124V and G224V, were found to be associated with skin cutaneous melanoma (SKCM) by comparison across tools, and one nsSNP, R86P, was found to be associated with ovarian serous cystadenocarcinoma (OV). However, no additional nsSNPs were detected from the mutation profiles supplied by this web server.

### 3.8. Analyzing the Gene Expression Profile for the PDCD1 Gene in Association with Cancer and Prognosis

**Figure 4:**
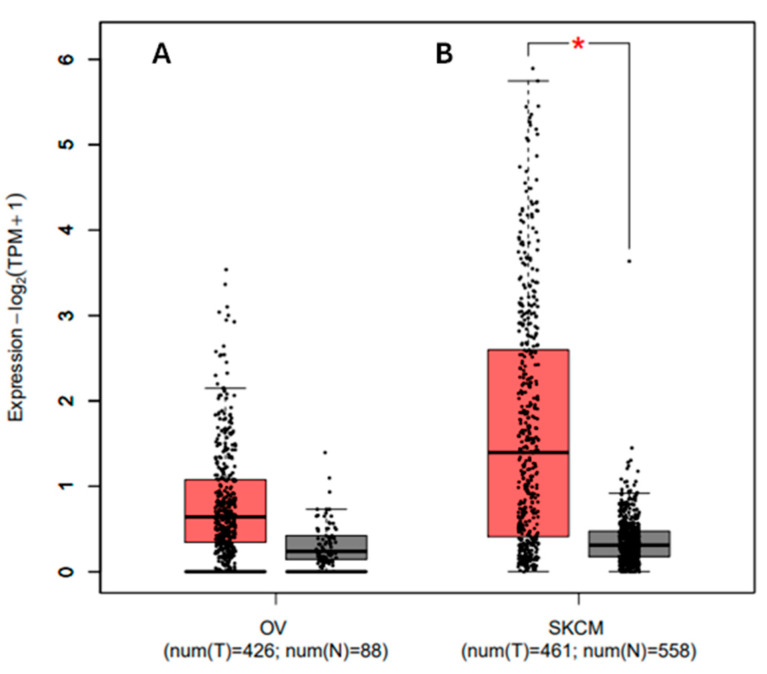
Differential expression of *PDCD1* in (**A**) ovarian cancer (OV) and (**B**) skin cutaneous melanoma (SKCM). Expression levels are shown as log2(TPM + 1) for tumor (T) and normal (N) samples. Significant upregulation of *PDCD1* was observed in SKCM tumor tissues compared to normal tissues (* *p* < 0.05), while differences in OV were less pronounced.

Using the GEPIA2 database, boxplots were produced to analyze the expression of the *PDCD1* gene and identify its differential expression patterns across different types of cancer. The expression of the *PDCD1* gene in ovarian cancer (OV) and skin cutaneous melanoma (SKCM) is shown in Figure 4 for tumor (T) and normal (N) tissue samples. The expression levels are shown as log2(TPM + 1) values for 426 tumors and 88 normal samples in OV, and for 461 tumors and 558 normal samples in SKCM.

In OV, the expression levels of *PDCD1* are relatively low in both tumor and normal samples, with overlapping interquartile ranges and no statistically significant differences. This suggests that *PDCD1* may not regulate immune checkpoint modulation in OV tumorigenesis.

In SKCM, however, the expression of *PDCD1* is significantly higher in tumor samples than in normal samples (*p* < 0.05), as indicated by the red asterisk. The median expression in tumor tissues is elevated, with greater variability and a wider range of values than in normal tissues. This upregulation suggests that *PDCD1* may be involved in immune checkpoint regulation and immune evasion mechanisms in SKCM. The boxplots further highlight these differences, with the red boxes representing tumor samples and the gray boxes representing normal samples.

### 3.9. Survival Analysis in OV and SKCM Patients

#### OV Patients

**Figure 5:**
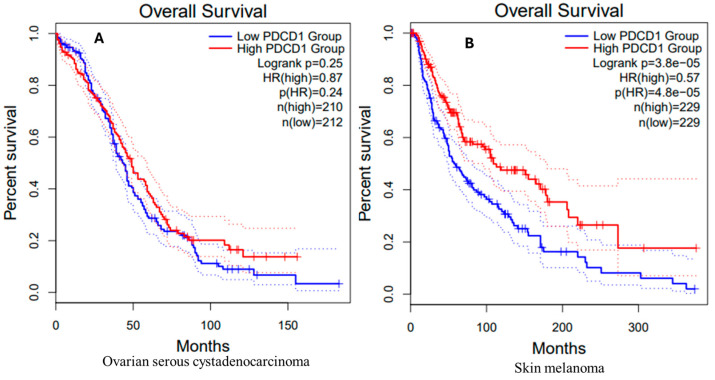
Kaplan–Meier survival curves of (**A**) ovarian serous cystadenocarcinoma (OV) and (**B**) skin cutaneous melanoma (SKCM) patients stratified by *PDCD1* expression levels. In OV, the red line represents the high-*PDCD1*-expression group (*n* = 210), and the blue line represents the low-expression group (*n* = 212). No significant survival difference was observed between the two groups (log-rank *p* = 0.25; HR = 0.87; *p*(HR) = 0.24). The dotted lines denote the 95% confidence intervals for each group. In SKCM, the red line represents the high-*PDCD1*-expression group (*n* = 229), and the blue line represents the low-expression group (*n* = 229). High *PDCD1* expression was significantly associated with improved overall survival (log-rank *p* = 3.8 × 10^−5^; HR = 0.57; *p*(HR) = 4.8 × 10^−5^). The dotted lines similarly indicate the 95% confidence intervals for each group.

Figure 5A illustrates Kaplan–Meier survival curves for overall survival in OV patients, categorized based on *PDCD1* expression levels. The *y*-axis shows percent survival (0% to 100%), and the *x*-axis shows survival time (months). Low and high *PDCD1* expressions are denoted by blue and red, respectively. The dotted lines show confidence intervals for variability in survival estimates.

Both curves show decreasing probabilities of survival over time. There is a slight improvement in survival in cases with high *PDCD1* expression (red curve) compared to those with low *PDCD1* expression (blue curve); however, this difference is not statistically significant (log-rank *p* = 0.25). The hazard ratio (HR) is 0.87, indicating a 13% reduced risk of death for the high-expression group, but this is not statistically significant (*p* = 0.24). The sample sizes are 210 for the high-expression group and 212 for the low-expression group.

#### SKCM Patients

The Kaplan–Meier survival curve for SKCM patients by high (red curve) and low (blue curve) *PDCD1* expression is shown in Figure 5B. The survival curves are surrounded by dotted lines denoting the confidence intervals of the survival estimates. Like OV, the *y*-axis is percent survival (0–100%) and the *x*-axis is the survival time in months.

In contrast to the observations in OV patients, SKCM patients with high *PDCD1* expression appear to have better survival outcomes than those with low expression since the probability of survival in the high-expression group declines slowly. Both the high- and low-expression groups contain 229 patients. There is a statistically significant difference in survival in cases with high *PDCD1* expression (red curve) compared to those with low *PDCD1* expression (blue curve), with a *p*-value of 3.8 × 10^−5^ (log-rank test, *p* < 0.05). The hazard ratio of 0.57 suggests that the risk of death is 43% lower in the high-expression group, a highly statistically significant result (*p* = 4.8 × 10^−5^). These results confirm a positive association between *PDCD1* expression levels and overall survival in SKCM patients.

### 3.10. Protein–Protein Interaction Analysis and Functional Enrichment Analysis

Using STRING software, we selected proteins that interact closely with the PD-1 protein. Our analysis showed that several key proteins are associated with PD-1, which is a critical inhibitory receptor on T cells that plays a central role in immune tolerance. PD-1 works by binding to its ligands CD274 (PD-L1) and CD273 (PD-L2) to initiate inhibitory signals that inhibit T-cell activation and restrict effector T-cell activity. Additionally, PDCD1LG2 is involved in costimulatory signals that promote T-cell proliferation and interferon-gamma (IFN-γ) production.

Other identified proteins, including CD80 and CD86, are involved in costimulatory signaling that is essential for T-cell activation. Furthermore, CD86 and CTLA4 are involved in negative regulatory functions for T cells, thereby further reducing immune responses. Furthermore, Galectin-9 (LGALS9) and Lymphocyte Activation Gene 3 (LAG3) were also identified as inhibitory molecules that transmit suppressive signals to T cells upon ligand binding.

**Figure 6:**
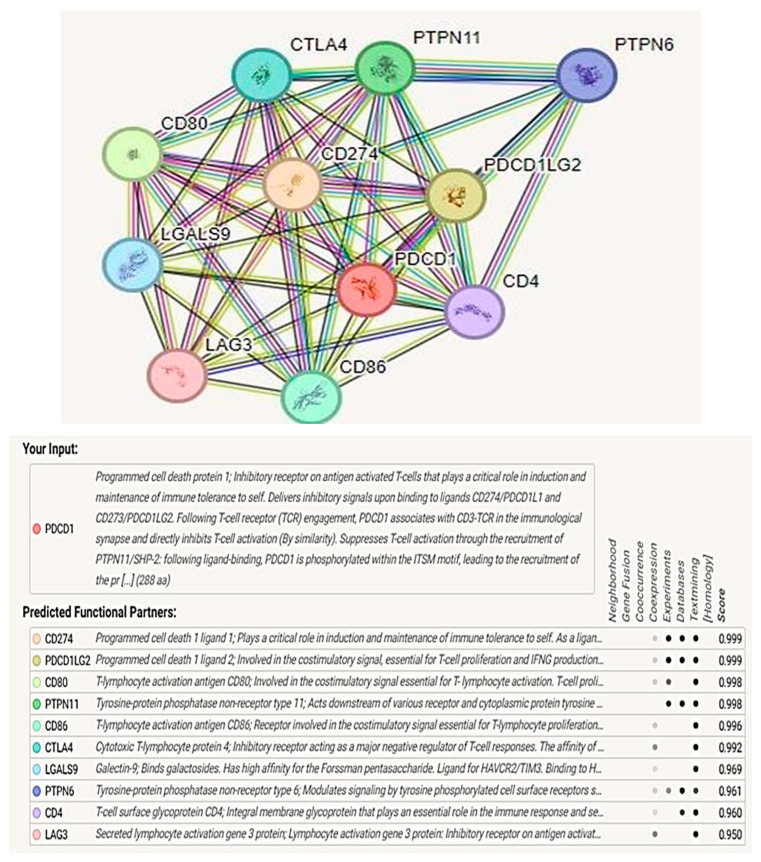
Protein–protein interaction network of *PDCD1* and its interacting partners, constructed using the STRING database. Key interacting proteins include CD274, CD80, CD86, CTLA4, LAG3, PTPN6, PTPN11, and LGALS9, among others. The network highlights the complex interactions between immune checkpoint molecules and signaling pathways, which play a crucial role in immune regulation. Colored lines between nodes represent various types of evidence for interactions, including known interactions (experimental and database-derived), predicted interactions, and text mining.

**Figure 7:**
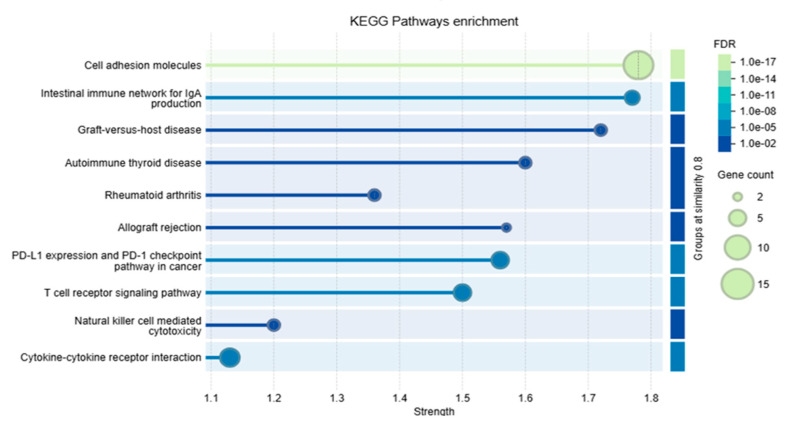
KEGG pathway enrichment analysis illustrating pathways (*y*-axis) ranked by their enrichment strength (*x*-axis). The size of each bubble represents the number of genes associated with the corresponding pathway, with larger bubbles indicating a higher gene count. The color gradient of the bubbles denotes the False Discovery Rate (FDR), reflecting statistical significance; darker colors correspond to lower FDR values (higher significance), while lighter colors indicate higher FDR values (lower significance). Horizontal bars behind the bubbles highlight the similarity between gene sets, with brighter bars representing greater similarity. Key pathways include “Cell adhesion molecules”, “Intestinal immune network for IgA production”, and others, showcasing their overrepresentation in the analysis.

**Figure 8:**
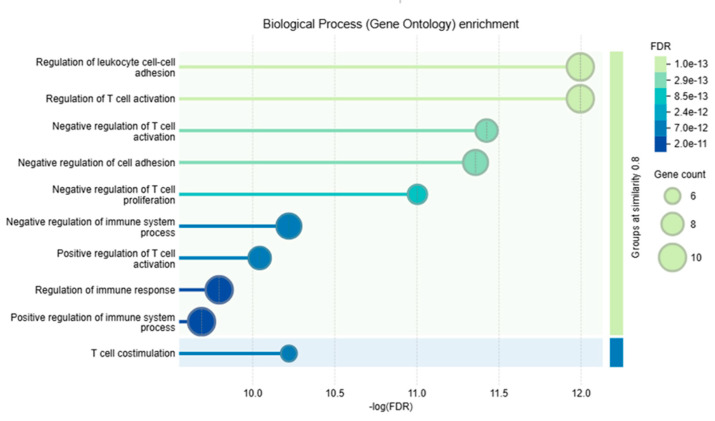
Biological Process (Gene Ontology) enrichment analysis showing key biological processes (*y*-axis) ranked by their statistical significance (−log (FDR) on the *x*-axis). Bubble size represents the gene count associated with each process, with larger bubbles indicating more genes involved. The color gradient of the bubbles reflects the False Discovery Rate (FDR), where darker colors denote lower FDR values (higher statistical significance) and lighter colors indicate higher FDR values (lower statistical significance). Horizontal bars behind the bubbles represent gene set similarity, with brighter bars indicating higher similarity among gene sets. Key processes include “Regulation of leukocyte cell-cell adhesion”, “Regulation of T-cell activation”, and “T-cell costimulation”, emphasizing their overrepresentation in the analysis.

Our analysis shows the role of tyrosine–protein phosphatases such as PTPN11 and PTPN6 that regulate signaling pathways that are involved in T-cell activation and hematopoiesis. CD4 acts as a co-receptor for MHC class II molecules and is essential for T-cell activation. These proteins combine to play a critical role in the modulation of the immune response and the maintenance of immune homeostasis, as depicted in Figure 6. Furthermore, functional enrichments of the PD-1 network, including KEGG and GO, demonstrate that the PD-1 protein is involved in multiple immune signaling pathways, as illustrated in Figure 7 and Figure 8.

### 3.11. Evaluation of the Functional Consequences of Non-Coding SNPs Using RegulomeDB

This analysis evaluated the functional effects of non-coding SNPs within the 5′ and 3′ UTRs of the *PDCD1* gene, focusing on their regulatory and miRNA target impacts using the ENSEMBL, RegulomeDB, and PolymiRTS databases.

Our findings show the identification of strong regulatory effects in the 3′ UTR via RegulomeDB. SNPs such as rs543306494, rs560497981 and rs550396273 were ranked as 2a, 2b, and 2b, respectively, suggesting high regulatory potential, with rs543306494 showing interactions with DNA-binding proteins and altered regulatory motifs (Table 5).

In the 3′ UTR, PolymiRTS analysis predicted miRNA target disruptions, as seen in rs142909968, which affected miRNA targets like hsa-miR-1296-5p and hsa-miR-4512, with negative context scores (−0.135 to −0.148) indicating reduced miRNA-mediated regulation. SNPs were categorized into functional classes (D, C, O, N), with Class D SNPs such as rs55942126 demonstrating strong disruption by targeting hsa-miR-1233-5p, while Class O variants (e.g., rs142909968) showed observed binding with minor regulatory impact (Table 6). 

## 4. Discussion

The programmed cell death protein 1 (PD-1) is a critical immune checkpoint molecule that acts as a suppressor of T-cell activity by interacting with its ligands, PD-L1 and PD-L2 [2,66]. This pathway plays a critical role in regulating immune tolerance and avoiding autoimmune diseases; nonetheless, cancer cells often exploit the PD-1/PD-L1 pathway to escape from immunosurveillance [9,10,67]. Genetic alterations, including nsSNPs in the *PDCD1* gene, can affect PD-1’s structure and its ability to bind ligands and modulate the immune response. These alterations can have a profound effect on the effectiveness of PD-1/PD-L1 inhibitor-based immunotherapies.

This study employed an in silico analysis to determine how nsSNPs affect the structure, stability, and function of the PD-1 protein, with emphasis on the pathogenic and oncogenic effects. We determined how nsSNPs affect the structural flexibility and stability of the PD-1 protein through its effects on the conformational dynamics of the protein. This study also assessed the level of expression of the *PDCD1* gene across different cancer types and investigated its relationship with overall survival rates in cancer patients. Beyond the coding regions, our analysis includes the effects of 3′ UTR variants on microRNA-binding sites, as well as the identification of functional variants in the 5′ UTR that may influence gene expression. The molecular pathogenicity of these nsSNPs was further analyzed to understand their roles in disease mechanisms. To the best of our knowledge, this is the first computational analysis to extensively investigate the impact of the nsSNPs in coding and non-coding of the *PDCD1* gene’s function, structure, and stability.

Out of the 695 nsSNPs analyzed, 84 were classified as deleterious by the six prediction tools used: MAPP, PhD-SNP, PolyPhen-1, PolyPhen-2, SIFT, and SNAP, as integrated through PredictSNP. This approach enhances the reliability and accuracy of identifying deleterious variants by integrating multiple predictive methods. These methods collectively evaluate different factors, including evolutionary conservation, protein structure, and functional impact, to ensure robust and precise predictions [51].

To determine the localization of these variants within the PD-1 protein, we mapped the nsSNPs to the specific domains of PD-1 using the InterPro program. This analysis showed that the nsSNPs were distributed across the domains as follows: 59 nsSNPs were identified in the non-cytoplasmic domain of PD-1, and 21 in other important regions. Such nsSNPs may affect the ability of PD-1 to bind its ligands. Furthermore, 24 nsSNPs were found in the cytoplasmic domain, which may affect the signaling pathways that are crucial for the regulation of the immune system. One nsSNP was found in the transmembrane domain of the PD-1, and such a variant may impact protein structure, adhesion, and signaling properties.

We found that nsSNPs significantly impacted the stability of the PD-1 protein. Computational analyses using I-Mutant 2.0 and MUpro revealed that a substantial proportion of the analyzed nsSNPs were classified as destabilizing. Specifically, the identification of 78 destabilizing nsSNPs highlights the significant role of protein stability—quantified by Gibbs free energy—in determining protein folding, structure, and function.

Some destabilizing nsSNPs might affect the function of PD-1 as an immune checkpoint by interfering with its ability to bind to its ligands (PD-L1/PD-L2). The susceptibility of the IgV-set domain to destabilizing mutations is particularly significant, as this domain is critical for ligand binding.

The structural effects of the wild-type versus mutant amino acids in the PD-1 protein were evaluated using Project HOPE based on the variations in the residue size, charge, hydrophobicity, and potential interactions caused by 78 missense nsSNPs. Mutations often led to significant variations in the structure of the PD-1 protein, especially in the regions that were well conserved. Of the 78 missense nsSNPs, for instance, mutations such as L17P introduced smaller residues that disrupted critical interactions, while larger residues in mutations such as C54R caused steric hindrance and, thus, disrupted the stability of the structure.

Differences in hydrophobicity, particularly in conserved regions (e.g., C54R, V144E), resulted in alterations of hydrogen bonding and reduced the efficiency of folding, thus impairing the protein’s functional stability. The effects of the mutations on the folding and stability of the core structure were evident, depending on the residue. For instance, in core packing, larger residues such as C54R and L65Q were found to cause folding defects through spatial incompatibility and the loss of hydrophobic packing interactions. C54R, Y223H, and L226P were the highly conserved residues, and any mutation in these residues affected the structure severely through folding disorders [68,69]. In contrast, mutations in less conserved residues, such as D26G and N49H, primarily caused localized changes, with limited effects on global stability [68,69].

PD-1 is glycosylated at specific sites such as N49, N58, N74, and N116, affecting ligand binding, receptor stability, and T-cell activity [36,38]. More specifically, core-fucosylation of PD-1 at N49 and N74 is essential for PD-1 expression, and its inhibition using 2-fluoro-L-fucose (2F-Fuc) has been found to increase T-cell activation and provides a promising potential target for immune checkpoint blockage treatment [39]. Additionally, the modulation of cemiplimab’s efficacy by glycosylation at N58 underscores the therapeutic importance of glycan-dependent interactions [40]. MutPred2 analysis revealed that pathogenic nsSNPs disrupt PD-1’s function through various molecular mechanisms, including alterations in glycosylation and other PTMs. The loss of N-linked glycosylation at N58 and N116, as observed in variants such as C54R and N116K, compromises ligand interactions and PD-1 stability, while the gain of glycosylation sites, such as N116 in D117V, introduces new functional dynamics. Furthermore, alterations in post-translational modifications—for example, sulfation loss at Y223 (Y223S)—affect phosphorylation-dependent signaling within the ITSM domain and, thus, inhibit PD-1’s immunosuppressive activity [41,42].

This study emphasizes the critical distinction between driver and passenger nsSNPs in cancer biology. To determine the oncogenic potential of PD-1 nsSNPs, we employed computational tools such as Cscape and CScape Somatic, and we identified several high-potential driver mutations that could be relevant to tumor development. Driver SNPs are functional; they affect protein function and, thus, play a role in cancer progression, while passenger SNPs are neutral mutations and do not affect the course of the disease [46,47,48,49,70]. The key mutations L17P, C54R, A80P, and L226P were found to be highly oncogenic, indicating that they are functionally important in promoting cancer. Out of all of the mutations, L226P exhibited the highest oncogenic score, suggesting a strong role in driving tumor progression. Similarly, mutations such as F228S and C241W were identified as oncogenic drivers, showing that the residues are commonly affected by driver mutations. These findings follow the established role of driver SNPs in activating oncogenic pathways or affecting immune regulation, especially in genes that encode immune checkpoint proteins such as PD-1. In contrast, certain nsSNPs—for instance, D117V and W186G—were classified as passenger mutations despite exhibiting moderate oncogenic scores. These mutations likely reflect neutral variations with no direct contribution to cancer progression.

The protein–protein interaction (PPI) network analysis, conducted using STRING, demonstrated the regulatory role of PD-1 (*PDCD1*) and its ligands PD-L1 (CD274) and PD-L2 (PDCD1LG2) in the suppression of T-cell activation. This interaction is crucial for maintaining immune tolerance, with dysregulation linked to the development of cancer and autoimmune diseases. Particularly, when high-pathogenicity nsSNPs such as C54R, Y68D, and W67C are incorporated into the PPI network or the ligand-binding domains of PD-1, they can affect the interactions of PD-1 with the ligands PD-L1 and PD-L2. Some of the structural variations include loss of disulfide bonds and variations in the transmembrane regions, which may affect PD-1’s binding and inhibitory signaling, thus affecting immune checkpoint control. The nsSNPs in proteins like CD80, CD86, and CTLA4 can alter their interactions within the PPI network.

In ovarian serous cystadenocarcinoma (OV), the *PDCD1* expression levels showed no significant statistical difference between tumor and normal tissues. This suggests that PD-1 might not be a key immune regulator in the ovarian tumor microenvironment. Kaplan–Meier survival analysis confirmed that high *PDCD1* expression did not significantly affect overall survival in OV patients, indicating that PD-1 expression may not be a critical prognostic factor in OV and other immunoregulatory pathways, or that tumor-intrinsic factors may be more important in the regulation of immunity in this disease. In contrast, in skin cutaneous melanoma (SKCM), *PDCD1* expression was found to be overexpressed in the tumor tissues as compared to the normal tissues. This is consistent with the current literature that documents the function of the PD-1/PD-L1 pathway in melanoma’s immune escape [9,10,71]. Kaplan–Meier survival analysis for SKCM revealed a strong association between high *PDCD1* expression and improved overall survival, indicating a 43% reduction in the risk of death for the high-expression group. This underlines the possibility that PD-1 might be a good prognostic factor in SKCM and points to its importance in the context of immune checkpoint regulation. Such overexpression may be a part of the immune response and could be enhanced with the use of checkpoint blockers such as nivolumab and pembrolizumab [9]. The significant survival advantage associated with PD-1’s upregulation in SKCM reinforces its role as a therapeutic target, particularly in cancers where immune evasion mechanisms are prominent.

The analysis of nsSNPs in the *PDCD1* gene revealed cancer-specific associations, including G124V and G224V in SKCM, as well as R86P in OV. Our analysis using the HOPE tool showed that G224V was very conserved, whereas others were less conserved and, thus, may not play a role in tumorigenesis. The association of G224V with SKCM indicates that these variants may have a greater relevance to the role of PD-1 in melanoma. T

This study highlights the context-dependent regulation of *PDCD1* across cancer types. Although PD-1 expression is not a significant prognostic marker in OV, its overexpression in SKCM is associated with improved survival and highlights its role in immune checkpoint regulation. Identification of cancer-specific nsSNPs also provides more insights into genetic variations that affect PD-1’s function, which may have significance in the context of personalized cancer therapy. Further research should also include functional analysis of these nsSNPs and the incorporation of genetic and transcriptomic annotations to further understand the regulation of PD-1 in various cancer types. These findings support the concept that the therapeutic approaches should be based on the molecular characteristics of the tumor to increase the clinical efficacy of immune checkpoint blockers.

This study also focused on the crucial role of non-coding SNPs within the untranslated regions (UTRs) of the *PDCD1* gene in regulating gene expression and immune checkpoint functions. The results are in agreement with previous studies that have shown that SNPs present in various regions of a gene, including the promoter, exon, and UTRs, can significantly influence gene expression and disease susceptibility. Specifically, UTR SNPs affect the transcriptional and post-transcriptional processes—for example, transcription factor binding and miRNA-mediated regulation, as seen in other genes, including PD-L1 [24,25,26,27,28,31].

Our results highlight the findings on the regulatory role of SNPs in the 3′ UTR of PD-1. Some of the important variants, such as rs543306494 and rs560497981, were identified as having strong regulatory potential, supported by interactions with DNA-binding proteins and disrupted regulatory motifs. Additionally, PolymiRTS analysis revealed that SNPs, including rs142909968 and rs55942126, interfere with miRNA-binding sites, for example, hsa-miR-1296-5p and hsa-miR-1233-5p, leading to the reduction in miRNA-mediated regulation, consistent with altered post-transcriptional control. These disruptions parallel observations in the PD-L1 gene, where SNPs affecting miRNA interactions are linked to increased cancer susceptibility and poor prognosis [31].

Furthermore, 5′ UTR SNPs demonstrated weaker but notable regulatory potential by affecting transcription factor binding and chromatin states. For instance, rs55970948 had a moderate regulatory effect through the alteration of the binding motif of transcription regulation. This is consistent with earlier studies that have shown that promoter and 5′ UTR SNPs can regulate gene expression through altering the transcriptional activity, as observed with PD-1 rs36084323 (−606 A > G) in cancer progression [32,33,34].

These observations emphasize the predominant role of 3′ UTR SNPs in disrupting miRNA interactions and altering post-transcriptional regulation, while 5′ UTR SNPs primarily influence transcriptional processes. Given the significant association of PD-1 expression with immune checkpoint regulation and disease outcomes, these SNPs represent promising biomarkers for predicting disease susceptibility and progression. Variants like rs142909968 and rs55942126, which strongly disrupt miRNA binding, may serve as key targets for further investigation.

While our study offers valuable insights into the role of PD-1 nsSNPs in cancer susceptibility, it is subject to certain limitations. Firstly, computational tools provide probabilistic predictions rather than definitive conclusions, and they should be interpreted as supportive evidence rather than conclusive proof. Computational predictions alone are inadequate for determining the pathogenicity of a genetic variant. As a result, comprehensive variant classification necessitates additional clinical validation through functional assays, segregation analysis, and population-based studies to enhance accuracy and reliability. Furthermore, computational tools, particularly in the context of ncSNPs, incorporate allele frequency data specific to certain populations, with some variants already experimentally validated in databases such as PolymiRTS and RegulomeDB.

## 5. Conclusions

In conclusion, this study highlights critical insights into how the nsSNPs in coding and non-coding regions within the *PDCD1* gene can affect its structure, function, and regulation, indicating their important roles in immune checkpoint modulation and cancer susceptibility. Using bioinformatics tools, we identified pathogenic nsSNPs that destabilize the PD-1 structure and might interfere with ligand binding, thus affecting immune regulation. Similarly, regulatory SNPs in UTRs such as rs543306494, rs560497981 and rs550396273 were found to influence the transcription and post-transcription processes, thereby affecting the PD-1 expression and function. 

Our findings highlight that the roles of *PDCD1* expression and nsSNPs vary according to the cancer type. For instance, *PDCD1* upregulation in SKCM was associated with better overall survival while showing limited prognostic value in ovarian cancer.

This study also demonstrates how nsSNPs can affect the overall immunoregulatory networks. Genetic alterations in proteins such as PD-1, PD-L1, and other signaling proteins, such as phosphatases, could lead to dysregulation of the immune system and promote tumor immune escape. Identifying driver mutations and regulatory nsSNPs provides the basis for developing new immune checkpoint inhibitor therapies.

While computational predictions offer robust insights, experimental validation will be crucial to confirm these findings and translate them into clinical applications. Future research should emphasize functional testing and combine the multi-omics data to optimize the therapeutic approaches targeting PD-1 and its associated pathways.

## Figures and Tables

**Table 1:** High-risk missense nsSNPs in the PD-1 protein.

SNP ID	AA Change	PredictSNP	MAPP, PhD-SNP, PolyPhan-1, PolyPhan-2, SIFT, SNAP
rs2124872179	L17P	Deleterious	Deleterious
rs1192883440	L25V	Deleterious	Deleterious
rs2124861799	D26G	Deleterious	Deleterious
rs2124861799	D26V	Deleterious	Deleterious
rs756045758	S27F	Deleterious	Deleterious
rs756045758	S27Y	Deleterious	Deleterious
rs1700939528	D29H	Deleterious	Deleterious
rs1017421889	D29V	Deleterious	Deleterious
rs1380273970	R30C	Deleterious	Deleterious
rs1044516789	R30H	Deleterious	Deleterious
rs1412459900	R30M	Deleterious	Deleterious
rs1412459900	R30T	Deleterious	Deleterious
rs751727384	P31L	Deleterious	Deleterious
rs757336262	P31T	Deleterious	Deleterious
rs2124861485	L42H	Deleterious	Deleterious
rs1700937498	G47R	Deleterious	Deleterious
rs2124861379	G47V	Deleterious	Deleterious
rs2124861330	N49H	Deleterious	Deleterious
rs2124861330	N49Y	Deleterious	Deleterious
rs2124861291	A50D	Deleterious	Deleterious
rs2124861238	C54R	Deleterious	Deleterious
rs2124861138	N58I	Deleterious	Deleterious
rs2124861011	L65P	Deleterious	Deleterious
rs2124861011	L65Q	Deleterious	Deleterious
rs1700935524	W67C	Deleterious	Deleterious
rs2124860994	W67R	Deleterious	Deleterious
rs2124860964	Y68D	Deleterious	Deleterious
rs2124860846	N74T	Deleterious	Deleterious
rs2124860823	Q75E	Deleterious	Deleterious
rs2124860779	D77H	Deleterious	Deleterious
rs2124860762	K78Q	Deleterious	Deleterious
rs987449655	L79V	Deleterious	Deleterious
rs2124860722	A80G	Deleterious	Deleterious
rs2124860734	A80P	Deleterious	Deleterious
rs2124860734	A80S	Deleterious	Deleterious
rs2124860734	A80T	Deleterious	Deleterious
rs1358028393	A81P	Deleterious	Deleterious
rs1358028393	A81T	Deleterious	Deleterious
rs1380350073	A81V	Deleterious	Deleterious
rs2124860682	F82S	Deleterious	Deleterious
rs1380273970	R86C	Deleterious	Deleterious
rs1044516789	R86P	Deleterious	Deleterious
rs1214961588	P89R	Deleterious	Deleterious
rs1700932618	D92G	Deleterious	Deleterious
rs1700932618	D92V	Deleterious	Deleterious
rs1427055411	R94C	Deleterious	Deleterious
rs757156727	R94H	Deleterious	Deleterious
rs757156727	R94P	Deleterious	Deleterious
rs757156727	R94L	Deleterious	Deleterious
rs758277335	R96C	Deleterious	Deleterious
rs773349951	R96P	Deleterious	Deleterious
rs533395656	V97F	Deleterious	Deleterious
rs2124860396	N102I	Deleterious	Deleterious
rs1256572186	N116K	Deleterious	Deleterious
rs772130993	D117V	Deleterious	Deleterious
rs2124860218	G119D	Deleterious	Deleterious
rs1230474759	G119S	Deleterious	Deleterious
rs2124860176	G124V	Deleterious	Deleterious
rs2124860004	V144E	Deleterious	Deleterious
rs1700919273	W186G	Deleterious	Deleterious
rs2124856625	D222Y	Deleterious	Deleterious
rs2124856605	Y223C	Deleterious	Deleterious
rs2124856613	Y223H	Deleterious	Deleterious
rs2124856613	Y223N	Deleterious	Deleterious
rs2124856605	Y223S	Deleterious	Deleterious
rs2124856588	G224V	Deleterious	Deleterious
rs2124856554	L226P	Deleterious	Deleterious
rs2124856554	L226Q	Deleterious	Deleterious
rs2124856517	F228C	Deleterious	Deleterious
rs2124856528	F228I	Deleterious	Deleterious
rs2124856528	F228L	Deleterious	Deleterious
rs2124856517	F228S	Deleterious	Deleterious
rs2124856517	F228Y	Deleterious	Deleterious
rs2124856503	Q229K	Deleterious	Deleterious
rs2124856279	C241W	Deleterious	Deleterious
rs2124856283	C241R	Deleterious	Deleterious
rs2124856201	Y248S	Deleterious	Deleterious
rs2124856181	A249D	Deleterious	Deleterious
rs2124856190	A249T	Deleterious	Deleterious
rs2124856153	I251T	Deleterious	Deleterious
rs2124856153	I251N	Deleterious	Deleterious
rs2124856129	V252D	Deleterious	Deleterious
rs2124856114	F253I	Deleterious	Deleterious
rs775100301	W286G	Deleterious	Deleterious

Note: The table presents a list of missense nsSNPs, identified by their respective SNP IDs. The “A.A change” column denotes the amino acid change resulting from each SNP. The “PredictSNP” column categorizes the predicted impact of each variant on protein function. The final column indicates the consensus classification of multiple computational prediction tools, including MAPP, PhD-SNP, PolyPhen-1, PolyPhen-2, SIFT, and SNAP.

**Table 2:** Effect of A.A change on *PD-1* protein stability according to I-Mutant 2.0 and MUpro.

SNP ID	AA Change	I-Mutant	RI	DDG-Free Energy Change Value (kcal/mol)	MUpro	DDG
rs2124872179	L17P	Decrease	4	−0.72	Decrease	−1.31
rs1192883440	L25V	Decrease	6	−0.05	Decrease	−0.85
rs2124861799	D26G	Decrease	2	−0.24	Decrease	−1.53
rs2124861799	D26V	Decrease	0	−0.17	Decrease	−0.43
rs756045758	S27F	Increase	1	−0.12	Decrease	−0.06
rs756045758	S27Y	Increase	3	−0.3	Decrease	−0.37
rs1700939528	D29H	Decrease	5	−0.79	Decrease	−0.89
rs1017421889	D29V	Decrease	1	−0.26	Decrease	−0.33
rs1380273970	R30C	Decrease	4	−0.94	Decrease	−0.77
rs1044516789	R30H	Decrease	7	−1.18	Decrease	−1.13
rs1412459900	R30M	Decrease	7	−2.11	Decrease	−0.49
rs1412459900	R30T	Decrease	8	−1.91	Decrease	−1.02
rs751727384	P31L	Decrease	5	−0.21	Decrease	−0.37
rs757336262	P31T	Decrease	9	−1.89	Decrease	−1.44
rs2124861485	L42H	Decrease	8	−2.2	Decrease	−1.79
rs1700937498	G47R	Decrease	8	−2.06	Decrease	−0.79
rs2124861379	G47V	Decrease	1	−0.88	Decrease	−0.52
rs2124861330	N49H	Decrease	8	−1.45	Decrease	−0.56
rs2124861330	N49Y	Decrease	1	0.1	Decrease	−0.34
rs2124861291	A50D	Decrease	0	−0.28	Decrease	−0.82
rs2124861238	C54R	Decrease	6	−1.15	Decrease	−1.05
rs2124861138	N58I	Increase	2	1.25	Decrease	−0.32
rs2124861011	L65P	Decrease	7	−0.34	Decrease	−2.04
rs2124861011	L65Q	Decrease	9	−1.34	Decrease	−1.63
rs1700935524	W67C	Decrease	7	−1.4	Decrease	−0.88
rs2124860994	W67R	Decrease	9	−1.65	Decrease	−0.82
rs2124860964	Y68D	Decrease	4	−0.29	Decrease	−1.66
rs2124860846	N74T	Decrease	0	−1.35	Decrease	−1.55
rs2124860823	Q75E	Increase	4	0.48	Decrease	−0.79
rs2124860779	D77H	Decrease	6	−0.57	Decrease	−1.21
rs2124860762	K78Q	Decrease	4	−0.76	Decrease	−0.23
rs987449655	L79V	Decrease	8	−0.54	Decrease	−0.94
rs2124860722	A80G	Decrease	8	−1.15	Decrease	−1.13
rs2124860734	A80P	Decrease	0	−1.52	Decrease	−0.89
rs2124860734	A80S	Decrease	9	−0.58	Decrease	−0.6
rs2124860734	A80T	Decrease	8	−1.08	Decrease	−0.73
rs1358028393	A81P	Decrease	1	−1.83	Decrease	−1.94
rs1358028393	A81T	Decrease	8	−1.2	Decrease	−1.55
rs1380350073	A81V	Decrease	2	−0.27	Decrease	−1.27
rs2124860682	F82S	Decrease	9	−2.07	Decrease	−2.37
rs1380273970	R86C	Decrease	5	−0.36	Decrease	−0.22
rs1044516789	R86P	Decrease	7	−1.89	Decrease	−0.74
rs1214961588	P89R	Decrease	7	−0.41	Decrease	−0.79
rs1700932618	D92G	Decrease	2	−0.93	Decrease	−0.78
rs1700932618	D92V	Increase	0	−0.2	Increase	0.07
rs1427055411	R94C	Decrease	5	−0.41	Decrease	−1.31
rs757156727	R94H	Decrease	8	−0.73	Decrease	−1.59
rs757156727	R94L	Decrease	8	−0.3	Decrease	−0.72
rs757156727	R94P	Decrease	5	−1.19	Decrease	−1.79
rs758277335	R96C	Decrease	5	−0.41	Decrease	−0.78
rs773349951	R96P	Decrease	5	−1.19	Decrease	−1.19
rs533395656	V97F	Decrease	8	−1.21	Decrease	−0.92
rs2124860396	N102I	Decrease	2	−0.01	Decrease	−0.02
rs1256572186	N116K	Decrease	7	−2.14	Decrease	−1.81
rs772130993	D117V	Decrease	5	−1.59	Decrease	−0.73
rs2124860218	G119D	Decrease	9	−1.22	Decrease	−0.43
rs1230474759	G119S	Decrease	9	−1.49	Decrease	−0.72
rs2124860176	G124V	Decrease	4	−1	Decrease	−0.28
rs2124860004	V144E	Decrease	4	−1.15	Decrease	−0.94
rs1700919273	W186G	Decrease	9	−2.7	Decrease	−1.54
rs2124856625	D222Y	Decrease	3	−0.77	Decrease	−0.89
rs2124856605	Y223C	Decrease	3	0.53	Decrease	−1.23
rs2124856613	Y223H	Decrease	8	−1.56	Decrease	−1.64
rs2124856613	Y223N	Decrease	6	−1.41	Decrease	−1.37
rs2124856605	Y223S	Decrease	8	−1.71	Decrease	−1.47
rs2124856588	G224V	Decrease	4	−0.82	Decrease	−0.67
rs2124856554	L226P	Decrease	7	−1.83	Decrease	−1.77
rs2124856554	L226Q	Decrease	9	−2.31	Decrease	−1.51
rs2124856517	F228C	Decrease	7	−2.06	Decrease	−1.1
rs2124856528	F228I	Decrease	7	−1.19	Decrease	−0.54
rs2124856528	F228L	Decrease	8	−2.08	Decrease	−0.58
rs2124856517	F228S	Decrease	9	−2.99	Decrease	−1.32
rs2124856517	F228Y	Decrease	4	−0.28	Decrease	−0.87
rs2124856503	Q229K	Increase	3	−0.02	Decrease	−1.74
rs2124856279	C241W	Decrease	3	−0.43	Decrease	−0.68
rs2124856283	C241R	Decrease	3	−0.87	Decrease	−0.62
rs2124856201	Y248S	Decrease	6	−1.33	Decrease	−1.41
rs2124856181	A249D	Decrease	2	−0.22	Decrease	−0.64
rs2124856190	A249T	Decrease	6	−0.74	Decrease	−0.85
rs2124856153	I251T	Decrease	8	−2.46	Decrease	−1.54
rs2124856153	I251N	Decrease	4	−0.95	Decrease	−1.38
rs2124856129	V252D	Decrease	8	−1.38	Decrease	−1.57
rs2124856114	F253I	Decrease	6	−0.71	Decrease	−0.55
rs775100301	W286G	Decrease	9	−2.5	Decrease	−2.03

Note: The table shows missense nsSNPs with their corresponding amino acid (AA) changes and predicted effects on protein stability. “I-Mutant” indicates whether the mutation decreases or increases protein stability, while “RI” represents the reliability index of the I-Mutant prediction. “DDG-Free Energy Change Value (kcal/mol)” reflects the predicted change in Gibbs free energy upon mutation, with negative values indicating destabilization. The “MUpro” column provides a secondary prediction of protein stability change, and “DDG” presents the associated stability change value. Decreased stability suggests potential structural and functional impacts on the protein.

**Table 3:** The results of MutPred2 software analysis of PD-1 nsSNPs, including MutPred2 scores and their impact on different molecular mechanisms.

AA Variation	MutPred2 Score	Molecular Mechanisms with *p*-Values Less than 0.05	*p*-Value
L17P	0.739	- Gain of loop	0.02
		- Altered transmembrane protein	0.02
C54R	0.962	- Altered metal binding	2.60 × 10^−3^
		- Loss of disulfide linkage at C54	5.50 × 10^−4^
		- Altered ordered interface	0.02
		- Altered transmembrane protein	5.60 × 10^−4^
		- Gain of strand	0.05
		- Loss of N-linked glycosylation at N58	5.6 × 10^−3^
		- Gain of catalytic site at C54	0.02
		- Gain of GPI-anchor amidation at N49	0.01
L65P	0.905	- Altered transmembrane protein	1.00 × 10^−4^
		- Altered stability	0.01
L65Q	0.827	- Altered transmembrane protein	7.80 × 10^−5^
		- Altered stability protein	0.05
W67C	0.844	- Altered transmembrane protein	1.20 × 10^−4^
		- Gain of intrinsic disorder	0.04
		- Loss of strand	1.70 × 10^−3^
		- Altered ordered interface	5.50 × 10^−3^
		- Loss of loop	0.04
		- Gain of disulfide linkage at W67	0.04
W67R	0.853	- Gain of intrinsic disorder	3.90 × 10^−3^
		- Altered ordered interface	0.04
		- Loss of strand	4.40 × 10^−3^
		- Altered transmembrane protein	3.90 × 10^−4^
		- Loss of loop	0.05
Y68D	0.925	- Gain of intrinsic disorder	7.90 × 10^−3^
		- Altered ordered interface	6.60 × 10^−3^
		- Altered transmembrane protein	6.50 × 10^−4^
		- Loss of strand	0.01
		- Altered stability	0.01
A80P	0.794	- Gain of intrinsic disorder	9.20 × 10^−3^
		- Altered transmembrane protein	1.50 × 10^−4^
		- Gain of strand	0.01
A81P	0.633	- Gain of intrinsic disorder	8.50 × 10^−3^
		- Altered stability	4.10 × 10^−3^
		- Altered transmembrane protein	1.50 × 10^−4^
		- Gain of strand	0.01
D117V	0.805	- Gain of ADP-ribosylation at R112	0.02
		- Altered disordered interface	0.04
		- Altered transmembrane protein	0.02
		- Gain of N-linked glycosylation at N116	0.02
W186G	0.627	- Loss of helix	2.90 × 10^−3^
		- Altered ordered interface	3.70 × 10^−3^
		- Altered signal peptide	2.10 × 10^−3^
Y223N	0.631	- Altered disordered interface	7.40 × 10^−3^
		- Altered ordered interface	8.80 × 10^−3^
		- Loss of strand	0.01
		- Loss of phosphorylation at Y223	0.03
		- Loss of proteolytic cleavage at D222	0.02
		- Altered transmembrane protein	0.02
		- Loss of sulfation at Y223	3.60 × 10^−3^
Y223S	0.529	- Altered disordered interface	8.30 × 10^−3^
		- Gain of intrinsic disorder	0.02
		- Altered ordered interface	6.20 × 10^−3^
		- Loss of strand	0.03
		- Gain of proteolytic cleavage at D222	1.90 × 10^−3^
		- Altered transmembrane protein	0.02
		- Loss of sulfation at Y223	3.60 × 10^−3^
L226P	0.533	- Gain of intrinsic disorder	3.40 × 10^−3^
		- Altered disordered interface	0.04
		- Altered stability	0.01
		- Gain of proteolytic cleavage at D222	0.02
		- Altered transmembrane protein	0.02
		- Gain of sulfation at Y223	2.30 × 10^−3^
Y248S	0.52	- Gain of intrinsic disorder	1.00 × 10^−3^
		- Altered ordered interface	0.02
		- Gain of O-linked glycosylation at T250	0.02
		- Altered stability	0.04
		- Altered metal binding	0.01
		- Loss of pyrrolidone carboxylic acid at Q245	0.04
I251N	0.634	- Gain of intrinsic disorder	7.70 × 10^−3^
		- Altered stability	0.01
		- Altered metal binding	0.01
		- Gain of sulfation at Y248	0.03
V252D	0.503	- Gain of intrinsic disorder	0.01
		- Altered stability	0.01
		- Altered metal binding	0.01

Note: This table displays the results of the MutPred2 software analysis of PD-1 nsSNPs. It includes the following columns: AA Variation: The amino acid substitutions caused by the SNP. MutPred2 Score: A confidence score (ranging from 0 to 1) that indicates the likelihood of a deleterious mutation, with higher scores suggesting greater pathogenicity. Molecular Mechanisms with *p*-values Less than 0.05: The predicted molecular functional and structural changes induced by the mutation. This includes alterations in protein stability, secondary structure, binding sites, post-translational modifications, and disorder. *p*-Value: The statistical significance associated with each predicted molecular mechanism; *p*-values below 0.05 indicate a significant probability that the mutation impacts the specified mechanism. These results offer insights into the potential functional consequences of PD-1 mutations, which may affect protein behavior and contribute to disease mechanisms.

**Table 4:** The oncogenic nature of nsSNPs predicted using Cscape and CScape Somatic.

	AA		Cscape		CScape Somatic	
SNP ID	Change	Input	Coding Score	Message	Coding Score	Warning
rs2124872179	L17P	2,242800941,A,G	0.6021	Oncogenic	0.839552	Driver
rs1192883440	L25V	2,242800918,A,C	0.581048	Oncogenic	0.661509	Driver
rs2124861238	C54R	2,242795049,A,G	0.627091	Oncogenic	0.50485	Driver
rs2124861011	L65P	2,242795015,A,G	0.561139	Oncogenic	0.407533	Passenger
rs2124861011	L65Q	2,242795015,A,T	0.503205	Oncogenic	0.270022	Passenger
rs1700935524	W67C	2,242795008,C,A	0.68252	Oncogenic	0.341358	Passenger
rs2124860994	W67R	2,242795010,A,T	0.697808	Oncogenic	0.292476	Passenger
rs2124860964	Y68D	2,242795007,A,C	0.682908	Oncogenic	0.450469	Passenger
rs987449655	L79V	2,242794974,G,C	0.668644	Oncogenic	0.419805	Passenger
rs2124860722	A80G	2,242794970,G,C	0.626369	Oncogenic	0.606956	Driver
rs2124860734	A80S	2,242794971,C,A	0.626567	Oncogenic	0.312374	Passenger
rs2124860734	A80P	2,242794971,C,G	0.76545	Oncogenic	0.679769	Driver
rs2124860734	A80T	2,242794971,C,T	0.613367	Oncogenic	0.30405	Passenger
rs1358028393	A81P	2,242794968,C,G	0.75435	Oncogenic	0.781253	Driver
rs772130993	D117V	2,242794859, T,A	0.5334	Oncogenic	0.376371	Passenger
rs1700919273	W186G	2,242794386, A,C	0.568592	Oncogenic	0.386691	Passenger
rs2124856625	D222Y	2,242793413, C,A	0.633163	Oncogenic	0.250538	Passenger
rs2124856605	Y223C	2,242793409, T,C	0.632424	Oncogenic	0.488143	Passenger
rs2124856605	Y223S	2,242793409,T,G	0.613155	Oncogenic	0.497009	Passenger
rs2124856613	Y223H	2,242793410,A,G	0.71215	Oncogenic	0.539332	Driver
rs2124856613	Y223N	2,242793410,A,T	0.647973	Oncogenic	0.396041	Passenger
rs2124856588	G224V	2,242793406,C,A	0.57769	Oncogenic	0.297241	Passenger
rs2124856554	L226P	2,242793400,A,G	0.80086	Oncogenic	0.631527	Driver
rs2124856554	L226Q	2,242793400,A,T	0.685336	Oncogenic	0.376093	Passenger
rs2124856517	F228C	2,242793394,A,C	0.719549	Oncogenic	0.486534	Passenger
rs2124856517	F228S	2,242793394,A,G	0.730018	Oncogenic	0.656042	Driver
rs2124856517	F228Y	2,242793394,A,T	0.675506	Oncogenic	0.326339	Passenger
rs2124856528	F228L	2,242793395,A,G	0.675661	Oncogenic	0.436829	Passenger
rs2124856528	F228I	2,242793395,A,T	0.661021	Oncogenic	0.295953	Passenger
rs2124856279	C241W	2,242793354,A,C	0.662798	Oncogenic	0.823574	Driver

Note: This table presents the oncogenic predictions of PD-1 nsSNPs using Cscape and Cscape- Somatic: SNP ID: Unique reference SNP identifier (rsID). AA Change: Amino acid substitution resulting from the SNP. Input: Genomic position and nucleotide change. Cscape Coding Score: Probability score >0.5 indicating the likelihood of the SNP being oncogenic; higher scores suggest stronger oncogenic potential. Cscape Message: Classification of the SNP as oncogenic. CScape Somatic Coding Score: Probability score (0–1) indicating oncogenic potential in somatic mutations. CScape Somatic Warning: Classification of the SNP as a driver (oncogenic mutation contributing to cancer) or passenger (neutral mutation with no significant oncogenic role).

**Table 5:** RegulomeDB analysis of 3′ and 5′ UTR non-coding SNPs in the PD-1 gene, showing chromosomal locations, dbSNP IDs, regulatory ranks, and scores.

3UTR			
**Chromosome Location**	**dbSNP IDs**	**Rank**	**Score**
chr2:241850262..241850263	rs543306494	2a	1
chr2:241850175..241850176	rs560497981	2b	0.82852
chr2:241850400..241850401	rs550396273	2b	1
chr2:241850045..241850046	rs554459879	4	0.70497
chr2:241850245..241850246	rs55676463	4	0.60906
chr2:241850249..241850250	rs1559446557	4	0.60906
chr2:241850531..241850532	rs186922590	4	0.60906
chr2:241850656..241850657	rs569664740	4	0.60906
chr2:241850657..241850658	rs536846778	4	0.60906
chr2:241850661..241850662	rs558753231	4	0.60906
chr2:241850699..241850700	rs554320171	4	0.60906
chr2:241850787..241850788	rs543637140	4	0.60906
chr2:241850802..241850803	rs565450127	4	0.60906
chr2:241850832..241850833	rs532613262	4	0.74401
chr2:241850953..241850954	rs565440440	4	0.70497
chr2:241850993..241850994	rs1400745867	4	0.60906
5UTR			
**Chromosome Location**	**dbSNP IDs**	**Rank**	**Score**
chr2:241858839..241858840	rs55970948	4	0.60906
chr2:241858858..241858859	rs544843762	4	0.60906
chr2:241858863..241858864	rs199970743	4	0.60906
chr2:241858873..241858874	rs374271577	4	0.60906

Note: This table presents the RegulomeDB analysis of non-coding SNPs in the 3′and 5′ untranslated regions (UTRs) of the PD-1 gene, assessing their potential regulatory impact. Chromosome Location: Genomic position of the SNP. dbSNP IDs: Unique reference SNP identifier (rsID). Rank: Regulatory classification score, from 1a (highest evidence of regulatory function) to 6 (minimal evidence), indicating the likelihood of affecting gene regulation. Score: A probability score (0–1), with higher values suggesting stronger regulatory potential. Regulatory SNPs in UTRs may influence gene expression, RNA stability, and protein translation, contributing to disease susceptibility and functional variations in PD-1 expression. In RegulomeDB, only the SNPs with a ranking < 4 were considered.

**Table 6:** PolymiRTS analysis of *PDCD1* ncSNPs.

Location	dbSNP ID	Variant Type	Wobble Base Pair	Ancestral Allele	Allele	miR ID	Conservation	miRSite	Function Class	Context+ Score Change
242792211	rs142909968	INDEL	N	-	C	hsa-miR-1296-5p	2	ggGGCCCTAgtacc	O	−0.135
						hsa-miR-4512	2	ggGGCCCTAgtacc	O	−0.148
						hsa-miR-6895-5p	2	ggGGCCCTAgtacc	O	−0.146
242792225	rs41379345	SNP	Y	G	A	hsa-miR-214-5p	2	ACAGGCAttcccc	C	−0.101
						hsa-miR-6514-3p	2	ACAGGCAttcccc	C	−0.171
						hsa-miR-6811-3p	2	ACAGGCAttcccc	C	−0.105
242792254	rs55942126	SNP	N	C	C	hsa-miR-1233-5p	2	ggCTCCCACcagg	D	−0.105
						hsa-miR-4300	2	gGCTCCCAccagg	D	−0.132
						hsa-miR-4456	2	ggctcCCACCAGg	D	−0.078
						hsa-miR-541-3p	2	ggctCCCACCAgg	D	−0.092
						hsa-miR-5591-5p	2	gGCTCCCAccagg	D	−0.125
						hsa-miR-6090	2	gGCTCCCAccagg	D	−0.149
						hsa-miR-654-5p	2	ggctCCCACCAgg	D	−0.102
						hsa-miR-6726-5p	2	gGCTCCCAccagg	D	−0.125
						hsa-miR-6769a-5p	2	ggctCCCACCAgg	D	−0.102
						hsa-miR-6769b-5p	2	ggctCCCACCAgg	D	−0.102
						hsa-miR-6778-5p	2	ggCTCCCACcagg	D	−0.086
						hsa-miR-6827-5p	2	GGCTCCCAccagg	D	−0.348
						hsa-miR-920	2	gGCTCCCAccagg	D	−0.132
						hsa-miR-92a-2-5p	2	ggctCCCACCAgg	D	−0.08
					A	hsa-miR-505-5p	2	GGCTCCAaccagg	C	−0.144
						hsa-miR-6874-5p	2	gGCTCCAAccagg	C	−0.115
						hsa-miR-92a-1-5p	2	ggctCCAACCAgg	C	−0.071
242792292	rs56015708	SNP	N	C	C	hsa-miR-4506	2	tggaACCCATTcc	D	−0.154
242792398	rs55676463	SNP	Y	G	A	hsa-miR-6512-3p	2	GGCTGGAgttgac	C	−0.099
						hsa-miR-6720-5p	2	GGCTGGAgttgac	C	−0.099
						hsa-miR-6849-3p	2	GGCTGGAgttgac	C	−0.102
						hsa-miR-766-3p	2	gGCTGGAGttgac	C	−0.099
242792447	rs41428445	SNP	N	C	T	hsa-miR-4790-3p	2	acACCATTCggga	C	0.007
242792748	rs6605260	SNP	N	C	C	hsa-miR-7113-5p	2	gaaacgCCCTGGA	D	−0.078
					T	hsa-miR-665	2	gaaacgTCCTGGA	C	−0.043
242792754	rs55869797	SNP	Y	G	A	hsa-miR-450b-3p	2	cATCCCAAaacgc	C	−0.067
						hsa-miR-5089-5p	2	cATCCCAAaacgc	C	−0.028
						hsa-miR-5187-5p	2	CATCCCAaaacgc	C	−0.084
						hsa-miR-6728-5p	2	CATCCCAAaacgc	C	−0.213
						hsa-miR-769-3p	2	cATCCCAAaacgc	C	−0.057
242792772	rs55721013	SNP	N	C	T	hsa-miR-3663-3p	2	aGGTGCTCctggc	C	−0.175
242792831	rs41492945	SNP	Y	G	A	hsa-miR-760	2	cgcccCAGAGCCt	C	−0.059
242792851	rs6605259	SNP	Y	G	A	hsa-miR-6508-3p	2	ggcgccATGGCCC	C	−0.092
242792926	rs111422100	SNP	Y	G	G	hsa-miR-4514	2	CTGCCTGcgtcca	D	−0.076
						hsa-miR-4692	2	CTGCCTGcgtcca	D	−0.085
					A	hsa-miR-1910-3p	2	CTGCCTAcgtcca	C	−0.084
						hsa-miR-2682-5p	2	CTGCCTAcgtcca	C	−0.114
						hsa-miR-34b-5p	2	CTGCCTAcgtcca	C	−0.11
						hsa-miR-449c-5p	2	CTGCCTAcgtcca	C	−0.121
						hsa-miR-6511a-5p	2	CTGCCTAcgtcca	C	−0.094
						hsa-miR-6808-5p	2	CTGCCTAcgtcca	C	−0.078
						hsa-miR-6893-5p	2	CTGCCTAcgtcca	C	−0.075
						hsa-miR-940	2	CTGCCTAcgtcca	C	−0.078
242793011	rs6749527	SNP	Y	G	G	hsa-miR-1304-3p	2	caCAGTGAGccca	D	−0.062
						hsa-miR-4284	3	cacaGTGAGCCca	D	−0.158

Note: This table presents the analysis of PD-1 gene variants and their potential impact on microRNA (miRNA)-binding sites, regulatory functions, and gene expression: Location: Chromosomal position of the SNP or insertion/deletion (INDEL). dbSNP ID: Unique reference SNP identifier (rsID). Variant Type: Type of mutation (SNP or INDEL). Wobble Base Pair: Indicates whether the SNP occurs in a wobble position, yes (Y) or no (N), affecting codon redundancy. Ancestral Allele: The original allele before mutation. Allele: The alternative allele introduced by the mutation. miR ID: The microRNA name predicted to interact with the SNP site. Conservation: Indicates evolutionary conservation of the SNP site (higher values suggest functional importance). miRSite: The predicted sequence context of the miRNA-binding site. Function Class: Polymorphic miRNA target sites are classified into four categories: “D” (the derived allele disrupts a conserved miRNA site), “N” (the derived allele disrupts a non-conserved miRNA site), “C” (the derived allele creates a new miRNA site), and “O” (cases where the ancestral allele cannot be determined). Context + Score Change: The predicted change in miRNA–mRNA interaction due to the SNP, where negative values suggest weakened binding and potential regulatory disruption. These findings help assess the impact of non-coding PD-1 variants on gene regulation through miRNA interactions, which may influence immune response and disease susceptibility.

## Data Availability

Data are contained within the article and Supplementary Materials.

## Supplementary Materials

The following supporting information can be downloaded at https://www.mdpi.com/article/10.3390/genes16030307/s1: Table S1: The percentage of high-risk nsSNPs in each program. Figure S1: Domain architecture of the PD-1 protein, showcasing its structural and functional features. Table S2: Interpretation of the impact of amino acid changes on PD-1’s protein structure and stability. Table S3: RegulomeDB variant classification scheme. Table S4: This table reports the allele frequency of seven nsSNPs in the coding region of *PDCD1* across genetic ancestry groups and sexes. Table S5: Allele frequency of SNPs in the 3′ untranslated region (3′UTR) of the *PDCD1* gene across different genetic ancestry groups and sexes.
