# Supplementary material for: Integrative In Silico Analysis to Identify Functional and Structural Impacts of nsSNPs on Programmed Cell Death Protein 1 (PD-1) Protein and UTRs: Potential Biomarkers for Cancer Susceptibility
Source: Genes (Basel). 2025 Mar 4;16(3):307. doi: 10.3390/genes16030307 (PMC11942535; doi:10.3390/genes16030307)
Supplement: Supplementary file 1 [file genes-16-00307-s001.zip › genes-3473206-supplementary.pdf]

Table S1: Percentage of high-risk PD-1 nsSNPs identified by each program.

SNP ID	AA change	PredictSNP	MAPP	PhD-SNP	PolyPhan-1	PolyPhan-2	SIFT	SNAP
**rs2124872179**	L17P	87%	78%	73%	74%	81%	79%	72%
**rs1192883440**	L25V	51%	84%	58%	67%	55%	67%	81%
**rs2124861799**	D26G	61%	86%	58%	59%	63%	46%	62%
**rs2124861799**	D26V	72%	84%	61%	59%	63%	53%	72%
**rs756045758**	S27F	65%	46%	68%	74%	43%	43%	72%
**rs756045758**	S27Y	65%	43%	68%	74%	54%	43%	72%
**rs1700939528**	D29H	53%	70%	72%	74%	43%	45%	62%
**rs1017421889**	D29V	61%	59%	59%	59%	61%	53%	58%
**rs1380273970**	R30C	72%	72%	66%	74%	65%	53%	72%
**rs1044516789**	R30H	65%	43%	72%	74%	65%	43%	72%
**rs1412459900**	R30M	65%	48%	78%	74%	60%	46%	81%
**rs1412459900**	R30T	55%	66%	68%	59%	43%	65%	62%
**rs751727384**	P31L	72%	77%	66%	74%	54%	79%	56%
**rs757336262**	P31T	72%	46%	58%	74%	50%	53%	62%
**rs2124861485**	L42H	87%	86%	68%	74%	81%	79%	85%
**rs1700937498**	G47R	87%	88%	73%	74%	81%	79%	85%
**rs2124861379**	G47V	87%	91%	59%	74%	81%	79%	85%
**rs2124861330**	N49H	87%	76%	59%	74%	65%	79%	56%
**rs2124861330**	N49Y	87%	76%	73%	74%	68%	79%	62%
**rs2124861291**	A50D	87%	88%	61%	74%	81%	79%	81%
**rs2124861238**	C54R	87%	88%	61%	74%	81%	79%	87%
**rs2124861138**	N58I	87%	72%	61%	74%	65%	79%	62%
**rs2124861011**	L65P	87%	88%	68%	74%	81%	79%	81%
**rs2124861011**	L65Q	87%	78%	68%	74%	81%	79%	81%
**rs1700935524**	W67C	87%	91%	77%	74%	81%	79%	89%
**rs2124860994**	W67R	87%	88%	77%	74%	81%	79%	87%
**rs2124860964**	Y68D	87%	86%	59%	74%	68%	45%	89%
**rs2124860846**	N74T	61%	63%	45%	59%	45%	53%	50%
**rs2124860823**	Q75E	51%	86%	78%	67%	59%	53%	50%
**rs2124860779**	D77H	55%	46%	83%	74%	56%	53%	50%
**rs2124860762**	K78Q	55%	77%	78%	67%	47%	53%	62%
**rs987449655**	L79V	64%	66%	89%	59%	60%	45%	81%
**rs2124860722**	A80G	64%	78%	89%	59%	54%	79%	56%
**rs2124860734**	A80P	72%	86%	68%	74%	81%	53%	62%
**rs2124860734**	A80S	65%	75%	72%	59%	50%	53%	62%
**rs2124860734**	A80T	55%	78%	72%	59%	63%	46%	61%
**rs1358028393**	A81P	72%	84%	68%	74%	68%	46%	62%
**rs1358028393**	A81T	61%	84%	78%	59%	63%	46%	50%
**rs1380350073**	A81V	55%	82%	83%	67%	54%	53%	56%
**rs2124860682**	F82S	76%	84%	55%	59%	81%	79%	62%
**rs1380273970**	R86C	55%	41%	83%	74%	68%	74%	81%
**rs1044516789**	R86P	55%	63%	66%	59%	55%	75%	72%
**rs1214961588**	P89R	55%	84%	78%	59%	47%	46%	50%

**rs1700932618**	D92G	52%	75%	72%	67%	40%	53%	56%
**rs1700932618**	D92V	76%	75%	58%	74%	56%	79%	62%
**rs1427055411**	R94C	76%	91%	55%	74%	81%	79%	89%
**rs757156727**	R94H	76%	77%	68%	74%	81%	79%	85%
**rs757156727**	R94L	76%	84%	68%	74%	81%	79%	85%
**rs757156727**	R94P	79%	88%	45%	74%	81%	79%	85%
**rs758277335**	R96C	76%	81%	66%	74%	81%	79%	72%
**rs773349951**	R96P	76%	86%	51%	74%	63%	46%	72%
**rs533395656**	V97F	76%	72%	58%	74%	55%	79%	72%
**rs2124860396**	N102I	87%	63%	68%	74%	55%	79%	56%
**rs1256572186**	N116K	87%	76%	82%	59%	50%	79%	56%
**rs772130993**	D117V	87%	77%	82%	74%	81%	79%	85%
**rs2124860218**	G119D	87%	88%	88%	74%	81%	79%	85%
**rs1230474759**	G119S	87%	57%	73%	74%	81%	79%	62%
**rs2124860176**	G124V	87%	91%	68%	74%	81%	79%	72%
**rs2124860004**	V144E	87%	92%	73%	74%	81%	79%	89%
**rs1700919273**	W186G	87%	84%	82%	59%	55%	79%	72%
**rs2124856625**	D222Y	87%	77%	59%	74%	81%	53%	72%
**rs2124856605**	Y223C	87%	82%	73%	74%	81%	79%	89%
**rs2124856613**	Y223H	87%	81%	59%	74%	81%	79%	85%
**rs2124856613**	Y223N	87%	86%	86%	74%	81%	79%	85%
**rs2124856605**	Y223S	87%	84%	86%	74%	81%	79%	85%
**rs2124856588**	G224V	87%	92%	59%	74%	68%	53%	72%
**rs2124856554**	L226P	87%	88%	82%	74%	81%	79%	81%
**rs2124856554**	L226Q	87%	78%	73%	74%	81%	79%	85%
**rs2124856517**	F228C	87%	86%	82%	74%	81%	79%	85%
**rs2124856528**	F228I	87%	72%	73%	74%	81%	79%	81%
**rs2124856528**	F228L	87%	75%	61%	59%	81%	79%	81%
**rs2124856517**	F228S	87%	84%	86%	74%	81%	79%	81%
**rs2124856517**	F228Y	87%	81%	59%	59%	81%	79%	81%
**rs2124856503**	Q229K	87%	82%	59%	59%	45%	79%	72%
**rs2124856279**	C241W	87%	63%	61%	74%	65%	79%	81%
**rs2124856283**	C241R	87%	88%	59%	74%	50%	79%	81%
**rs2124856201**	Y248S	87%	84%	77%	74%	81%	79%	81%
**rs2124856181**	A249D	87%	88%	61%	74%	81%	79%	85%
**rs2124856190**	A249T	87%	92%	58%	74%	81%	79%	72%
**rs2124856153**	I251T	87%	84%	73%	74%	81%	79%	72%
**rs2124856153**	I251N	87%	86%	77%	74%	81%	79%	81%
**rs2124856129**	V252D	87%	92%	61%	74%	81%	79%	89%
**rs2124856114**	F253I	87%	72%	58%	74%	81%	79%	81%
**rs775100301**	W286G	87%	82%	58%	74%	68%	79%	81%

Note: This table presents the computational predictions for the functional impact of missense non-synonymous single nucleotide polymorphisms (nsSNPs) using multiple in silico tools. These tools evaluate the potential pathogenicity of amino acid substitutions based on evolutionary conservation, structural properties, and biochemical changes.

- SNP ID: Reference identifier for each SNP.
- AA change: Amino acid substitution caused by the SNP.
- PredictSNP: a consensus predictor by integrating multiple prediction algorithms to estimate the probability of a deleterious effect. It combines six of the best prediction tools (MAPP, PhD-SNP, PolyPhen-1, PolyPhen-2, SIFT, and SNAP)
- MAPP: Assesses the impact of mutations based on evolutionary conservation and physicochemical differences between amino acids.
- PhD-SNP: Predicts whether a mutation is disease-associated using a machine-learning approach.
- PolyPhen-1 & PolyPhen-2: Analyze the potential impact of mutations on protein structure and function based on sequence and structural features.
- SIFT: Predicts whether an amino acid substitution is tolerated based on sequence homology and conservation.
- SNAP: Uses neural networks to classify mutations as functionally neutral or non-neutral.

The values in the table represent the probability (%) that the mutation is deleterious or functionally impactful, with higher percentages indicating a greater likelihood of pathogenicity. These predictions help prioritize variants for experimental validation and further functional studies.

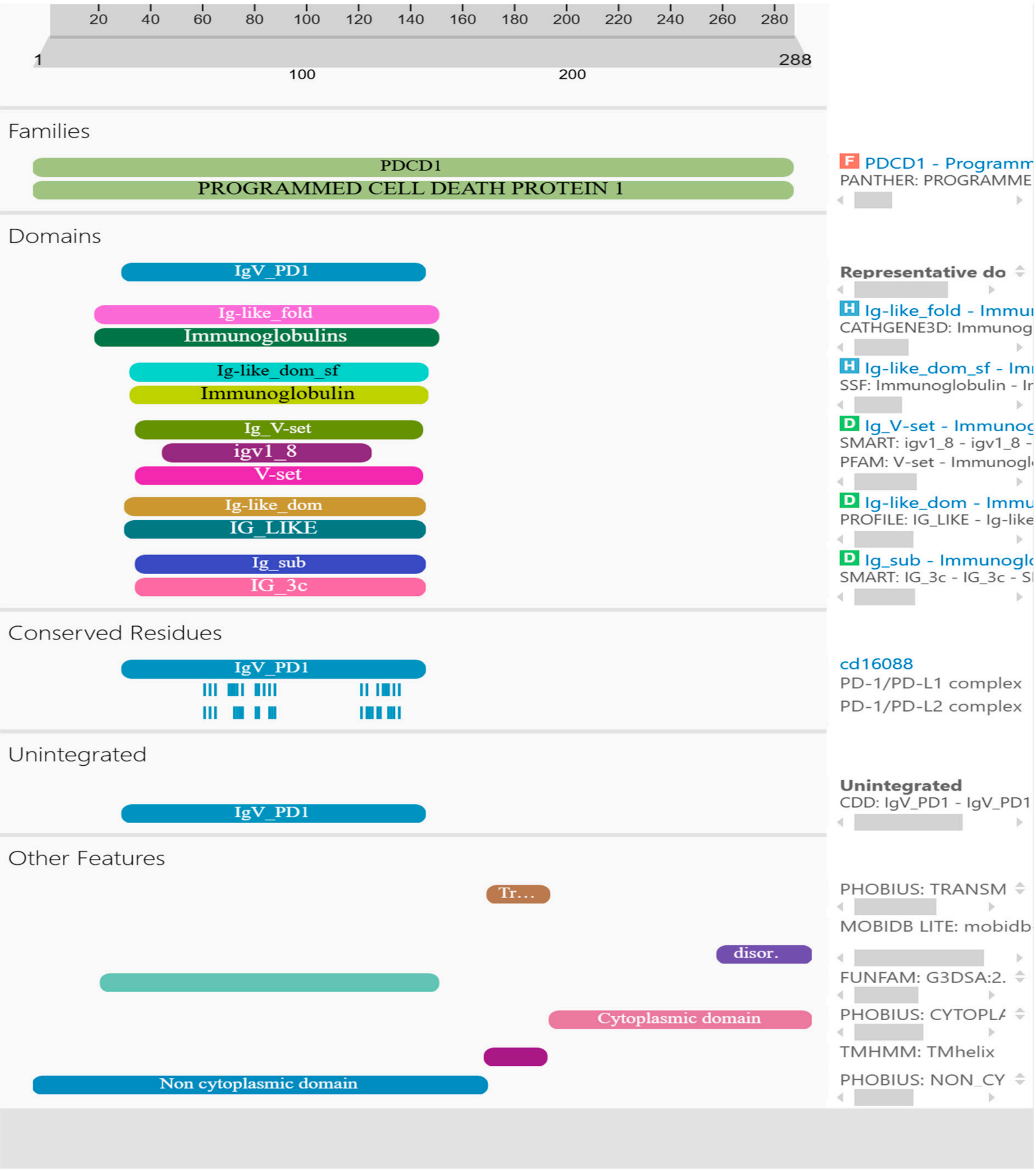

Figure S1: The figure illustrates the structural and functional annotation of Programmed Cell Death Protein 1 (PDCD1, PD-1) based on domain classification, conserved residues, and other features.

- Families: The PDCD1 protein is categorized under the Programmed Cell Death Protein 1 family.
- Domains: Multiple immunoglobulin-like (Ig-like) domains are present, including IgV PD1, Immunoglobulin, Ig-like fold, V-set, and IG_LIKE, indicating its role in immune regulation.
- Conserved Residues: The IgV PD1 domain contains highly conserved residues, essential for its structural stability and function.
- Unintegrated Domains: The IgV PD1 domain is displayed as an unintegrated feature in some annotation sources.
- Other Features: The protein contains:

- o Non-cytoplasmic domain (blue), indicating extracellular regions.
- o Cytoplasmic domain (pink), involved in intracellular signaling.
- o Disordered regions (purple), which may contribute to flexible interactions.
- o Transmembrane segment (TR) suggesting membrane localization.

These structural annotations provide insight into PD-1's role in immune checkpoint regulation, particularly in its interaction with PD-L1 and PD-L2 in immune signaling pathways.

Table S2: Interpretation of the impact of amino acid change on *PD-1* protein structure andstability.

SNP ID	AA change	AA Properties	Conservation
rs2124872179	L17P	✓ The wild-type and mutant amino acids differ in size.<br>✓ The mutant residue is smaller, this might lead to loss of interactions.	Very conserved
rs1192883440	L25V	✓ The wild-type and mutant amino acids differ in size.<br>✓ The mutant residue is smaller, this might lead to loss of interactions.<br>✓ The wild-type and mutant amino acids differ in size.<br>✓ The mutant residue is smaller, this might lead to loss of interactions.<br>✓ The hydrophobicity of the wild-type and mutant residue differs.<br>✓ The mutation introduces a more hydrophobic residue at this position.<br>✓ This can result in loss of hydrogen bonds and/or disturb correct folding.	Very conserved
rs2124861485	L42H	✓ The wild-type and mutant amino acids differ in size.<br>✓ The mutant residue is bigger than the wild-type residue.<br>✓ The residue is located on the surface of the protein, mutation of this residue can disturb interactions with other molecules or other parts of the protein.<br>✓ The hydrophobicity of the wild-type and mutant residue differs.<br>✓ The mutation might cause loss of hydrophobic interactions with other molecules on the surface of the protein.	Very conserved
rs1700937498	G47R	✓ There is a difference in charge between the wild-type and mutant amino acid.<br>✓ The mutation introduces a charge at this position, this can cause repulsion between the mutant residue and neighboring residues.<br>✓ The wild-type and mutant amino acids differ in size.<br>✓ The mutant residue is bigger than the wild-type residue.<br>✓ The residue is located on the surface of the protein, mutation of this residue can disturb interactions with other molecules or other parts of the protein.	100% conserved

		✓ The torsion angles for this residue are unusual. only glycine is flexible enough to make these torsion angles, mutation into another residue will force the local backbone into an incorrect conformation and will disturb the local structure.	
rs2124861379	G47V	✓ The wild-type and mutant amino acids differ in size.<br>✓ The mutant residue is bigger than the wild-type residue.<br>✓ The residue is located on the surface of the protein, mutation of this residue can disturb interactions with other molecules or other parts of the protein.	100% conserved
rs2124861238	C54R	✓ The torsion angles for this residue are unusual. only glycine is flexible enough to make these torsion angles, mutation into another residue will force the local backbone into an incorrect conformation and will disturb the local structure.<br>✓ There is a difference in charge between the wild-type and mutant amino acid.<br>✓ The mutant residue introduces a charge in a buried residue which can lead to protein folding problems.<br>✓ The wild-type and mutant amino acids differ in size.<br>✓ The mutant residue is bigger than the wild-type residue.<br>✓ The wild-type residue was buried in the core of the protein. The mutant residue is bigger and probably will not fit.<br>✓ The hydrophobicity of the wild-type and mutant residue differs.<br>✓ The mutation will cause loss of hydrophobic interactions in the core of the protein.	100% conserved
rs2124861011	L65P	✓ The wild-type and mutant amino acids differ in size.<br>✓ The mutant residue is smaller than the wild-type residue.<br>✓ The mutation will cause an empty space in the core of the protein.	Very conserved
rs2124861011	L65Q	✓ The wild-type and mutant amino acids differ in size.<br>✓ The mutant residue is bigger than the wild-type residue.<br>✓ The wild-type residue was buried in the core of the protein. The mutant residue is bigger and probably will not fit.<br>✓ The hydrophobicity of the wild-type and mutant residue differs.<br>✓ The mutation will cause loss of hydrophobic interactions in the core of the protein.	Very conserved
rs1700935524	W67C	✓ The wild-type and mutant amino acids differ in size.<br>✓ The mutant residue is smaller than the wild-type residue.<br>✓ The mutation will cause an empty space in the core of the protein.	Very conserved
rs2124860994	W67R	✓ There is a difference in charge between the wild-type and mutant amino acid.	Very conserved

		✓ The mutant residue introduces a charge in a buried residue which can lead to protein folding problems.<br>✓ The wild-type and mutant amino acids differ in size.<br>✓ The mutant residue is smaller than the wild-type residue.<br>✓ The mutation will cause an empty space in the core of the protein.<br>✓ The hydrophobicity of the wild-type and mutant residue differs.<br>✓ The mutation will cause loss of hydrophobic interactions in the core of the protein.	
rs2124860964	Y68D	✓ There is a difference in charge between the wild-type and mutant amino acid.<br>✓ The mutant residue introduces a charge in a buried residue which can lead to protein folding problems.<br>✓ The wild-type and mutant amino acids differ in size.<br>✓ The mutant residue is smaller than the wild-type residue.<br>✓ The mutation will cause an empty space in the core of the protein.<br>✓ The hydrophobicity of the wild-type and mutant residue differs.<br>✓ The mutation will cause loss of hydrophobic interactions in the core of the protein.	Very conserved
rs987449655	L79V	✓ The wild-type and mutant amino acids differ in size.<br>✓ The mutant residue is smaller than the wild-type residue.<br>✓ The mutation will cause an empty space in the core of the protein.	Very conserved
rs2124860722	A80G	✓ The wild-type and mutant amino acids differ in size.<br>✓ The mutant residue is smaller than the wild-type residue.<br>✓ This will cause a possible loss of external interactions.<br>✓ The hydrophobicity of the wild-type and mutant residue differs.<br>✓ The mutation might cause loss of hydrophobic interactions with other molecules on the surface of the protein.	Very conserved
rs2124860734	A80P	✓ The wild-type and mutant amino acids differ in size.<br>✓ The mutant residue is bigger than the wild-type residue.<br>✓ The residue is located on the surface of the protein, mutation of this residue can disturb interactions with other molecules or other parts of the protein.	Very conseíved
rs2124860734	A80S	✓ The wild-type and mutant amino acids differ in size.<br>✓ The mutant residue is bigger than the wild-type residue.<br>✓ The residue is located on the surface of the protein, mutation of this residue can disturb interactions with other molecules or other parts of the protein.	Very conserved

		✓ The hydrophobicity of the wild-type and mutant residue differs.<br>✓ The mutation might cause loss of hydrophobic	
rs2124860734	A80T	interactions with other<br>molecules on the surface of the protein.<br>✓ The wild-type and mutant amino acids differ in size.<br>✓ The mutant residue is<br>bigger than the wild-type residue.<br>✓ The residue is located on the surface of the protein, mutation of this residue can disturb interactions with other molecules or other parts of the protein.<br>✓ The hydrophobicity of the wild-type and mutant residue differs.<br>✓ The mutation might cause loss of hydrophobic interactions with other<br>molecules on the surface of the protein.	Very conserved
rs1700932618	D92G	✓ There is a difference in charge between the wild-type and mutant amino<br>acid.<br>✓ The charge of the wild-type residue will be lost, this can cause loss of interactions with other molecules or residues.<br>✓ The wild-type and mutant amino acids differ in size.<br>✓ The mutant residue is smaller, this might lead to loss of interactions.<br>✓ The hydrophobicity of the wild-type and mutant residue differs.<br>✓ The mutation introduces a more hydrophobic residue at this position. This can result in loss of hydrogen bonds and/or disturb correct folding.	Very conserved
rs772130993	D117V	✓ There is a difference in charge between the wild-type and mutant amino acid.<br>✓ The charge of the buried wild-type residue is lost by this mutation.<br>✓ The wild-type and mutant amino acids differ in size.<br>✓ The mutant residue is smaller than the wild-type residue.<br>✓ The mutation will cause an empty space in the core of the protein.<br>✓ The hydrophobicity of the wild-type and mutant residue differs.<br>✓ The mutation will cause loss of hydrogen bonds in the core of the protein and as a result disturb correct folding.	Very conserved
rs1700919273	W186G	✓ The wild-type and mutant amino acids differ in size.<br>✓ The mutant residue is smaller, this might lead to loss of interactions.<br>✓ The hydrophobicity of the wild-type and mutant residue differs.<br>✓ Hydrophobic interactions, either in the core of the protein or on the surface, will be lost.	Very conserved

rs2124856625	D222Y	✓ There is a difference in charge between the wild-type and mutant amino acid.<br>✓ The charge of the wild-type residue will be lost, this can cause loss of interactions with other molecules or residues.<br>✓ The wild-type and mutant amino acids differ in size.<br>✓ The mutant residue is<br>bigger, this might lead to bumps.<br>✓ The hydrophobicity of the wild-type and mutant residue differs.<br>✓ The mutation introduces a more hydrophobic residue at this position. This can result in loss of hydrogen bonds and/or disturb correct folding.	Very conserved
rs2124856605	Y223C	✓ The wild-type and mutant amino acids differ in size.<br>✓ The mutant residue is smaller, this might lead to loss of interactions.<br>✓ The hydrophobicity of the wild-type and mutant residue differs.<br>✓ The mutation introduces a more hydrophobic residue at this position. This can result in loss of hydrogen bonds and/or disturb correct folding.	100% conserved
rs2124856613	Y223H	✓ The wild-type and mutant amino acids differ in size.<br>✓ The mutant residue is smaller, this might lead to loss of interactions.<br>✓ The hydrophobicity of the wild-type and mutant residue differs.<br>✓ Hydrophobic interactions, either in the core of the protein or on the surface, will be lost.	100% conserved
rs2124856613	Y223N	✓ The wild-type and mutant amino acids differ in size.<br>✓ The mutant residue is smaller, this might lead to loss of interactions.<br>✓ The hydrophobicity of the wild-type and mutant residue differs.<br>✓ Hydrophobic interactions, either in the core of the protein or on the surface, will be lost.	100 % conserved
rs2124856605	Y223S	✓ The wild-type and mutant amino acids differ in size.<br>✓ The mutant residue is smaller, this might lead to loss of interactions.	100% conserved
rs2124856588	G224V	✓ The wild-type and mutant amino acids differ in size.<br>✓ The mutant residue is bigger, this might lead to bumps.<br>✓ The torsion angles for this residue are unusual. only glycine is flexible enough to make these torsion angles, mutation into another residue will force the local backbone into an incorrect conformation and will disturb the local structure.	Very conserved
rs2124856554	L226P	✓ The wild-type and mutant amino acids differ in size.<br>✓ The mutant residue is smaller, this might lead to loss of interactions.	100% conserved
rs2124856554	L226Q	✓ The wild-type and mutant amino acids differ in size.<br>✓ The mutant residue is bigger, this might lead to bumps.	100% conserved

		✓ The hydrophobicity of the wild-type and mutant residue differs.<br>✓ Hydrophobic interactions, either in the core of the protein or on the surface, will be lost.	
rs2124856517	F228C	✓ The wild-type and mutant amino acids differ in size.<br>✓ The mutant residue is smaller, this might lead to loss of interactions.	100% conserved
rs2124856528	F228I	✓ The wild-type and mutant amino acids differ in size.<br>✓ The mutant residue is smaller, this might lead to loss of interactions.	100 % conserved
rs2124856528	F228L	✓ The wild-type and mutant amino acids differ in size.<br>✓ The mutant residue is smaller, this might lead to loss of interactions.	100 % conserved
rs2124856517	F228S	✓ The wild-type and mutant amino acids differ in size.<br>✓ The mutant residue is smaller, this might lead to loss of interactions.<br>✓ The hydrophobicity of the wild-type and mutant residue differs.<br>✓ Hydrophobic interactions, either in the core of the protein or on the surface, will be lost.	100% conserved
rs2124856517	F228Y	✓ The wild-type and mutant amino acids differ in size.<br>✓ The mutant residue is bigger, this might lead to bumps.<br>✓ The hydrophobicity of the wild-type and mutant residue differs.<br>✓ Hydrophobic interactions, either in the core of the protein or on the surface, will be lost.	100 % conserved
rs2124856279	C241W	✓ The wild-type and mutant amino acids differ in size.<br>✓ The mutant residue is bigger, this might lead to bumps.	Very conserved
rs2124856283	C241R	✓ There is a difference in charge between the wild-type and mutant amino acid.<br>✓ The mutation introduces a charge, this can cause repulsion of ligands or other residues with the same charge.<br>✓ The wild-type and mutant amino acids differ in size.<br>✓ The mutant residue is bigger, this might lead to bumps.<br>✓ The hydrophobicity of the wild-type and mutant residue differs.<br>✓ Hydrophobic interactions, either in the core of the protein or on the surface, will be lost.	Very conserved
rs2124856201	Y248S	✓ The wild-type and mutant amino acids differ in size.<br>✓ The mutant residue is smaller, this might lead to loss of interactions.	Very conserved
rs2124856181	A249D	✓ There is a difference in charge between the wild-type and mutant amino acid.<br>✓ The mutation introduces a charge, this can cause repulsion of ligands or other residues with the same charge.<br>✓ The wild-type and mutant amino acids differ in size.<br>✓ The mutant residue is bigger, this might lead to bumps.	Very conserved

		✓ The hydrophobicity of the wild-type and mutant residue differs.<br>✓ Hydrophobic interactions, either in the core of the protein or on the surface, will be lost.	
rs2124856190	A249T	✓ The wild-type and mutant amino acids differ in size.<br>✓ The mutant residue is bigger, this might lead to bumps.<br>✓ The hydrophobicity of the wild-type and mutant residue differs.<br>✓ Hydrophobic interactions, either in the core of the protein or on the surface, will be lost.	Very conserved
rs2124856153	I251T	✓ The wild-type and mutant amino acids differ in size.<br>✓ The mutant residue is smaller, this might lead to loss of interactions.<br>✓ The hydrophobicity of the wild-type and mutant residue differs.<br>✓ Hydrophobic interactions, either in the core of the protein or on the surface, will be lost.	Very conserved
rs2124856153	I251N	✓ The wild-type and mutant amino acids differ in size.<br>✓ The mutant residue is bigger, this might lead to bumps.<br>✓ The hydrophobicity of the wild-type and mutant residue differs.<br>✓ Hydrophobic interactions, either in the core of the protein or on the surface, will be lost.	Very conserved
rs2124856129	V252D	✓ There is a difference in charge between the wild-type and mutant amino acid.<br>✓ The mutation introduces a charge, this can cause repulsion of ligands or other residues with the same charge.<br>✓ The wild-type and mutant amino acids differ in size.<br>✓ The mutant residue is bigger, this might lead to bumps.<br>✓ The hydrophobicity of the wild-type and mutant residue differs.<br>✓ Hydrophobic interactions, either in the core of the protein or on the surface, will be lost.	Very conserved
rs2124856114	F253I	✓ The wild-type and mutant amino acids differ in size.<br>✓ The mutant residue is smaller, this might lead to loss of interactions.	Very conserved
rs775100301	W286G	✓ The wild-type and mutant amino acids differ in size.<br>✓ The mutant residue is smaller, this might lead to loss of interactions.<br>✓ The hydrophobicity of the wild-type and mutant residue differs.<br>✓ Hydrophobic interactions, either in the core of the protein or on the surface, will be lost.	100% conserved

Note: this table provides detailed annotations of nSNPs and their potential impact on protein structure and function. The table includes information on

- SNP ID: Reference identifier for each SNP.
- Amino Acid (AA) Change: Substitution of the wild-type residue with the mutant residue.
- AA Properties: Biochemical and structural changes caused by the variant, including differences in size, charge, and hydrophobicity, potential effects on protein stability, interactions, and folding, as well as torsion angles and hydrogen bonding.

- Conservation: Evolutionary conservation of the residue, with "Very conserved" indicating strong evolutionary constraints and "100% conserved" suggesting absolute conservation across species, implying functional importance. Highly conserved residues often play critical structural or functional roles in proteins, and mutations at these positions are more likely to have deleterious effects. The information in this table aids in assessing the functional significance of these variants for further experimental validation.

Table S3. RegulomeDB variant classification scheme

Category	Description
	Likely to affect binding and linked to the expression of a gene target
1a	eQTL+ TF binding + matched TF motif + matched DNase footprint +DNase peak
1b	eQTL+ TF binding + any motif + DNase footprint +DNase peak
1c	eQTL+ TF binding + matched TF motif +DNase peak
1d	eQTL+ TF binding + any motif + DNase footprint +DNase peak
1e	eQTL+ TF binding + matched TF motif
1f	eQTL+ TF binding / DNase peak
	Likely to affect the binding
2a	TF binding + matched TF motif + matched DNase footprint +DNase peak
2b	TF binding + any motif +DNase footprint +DNase peak
2c	TF binding + matched TF motif +DNase peak
	Less likely to affect the binding
3a	TF binding + any motif +DNase peak
3b	TF binding + matched TF motif
	Minimal binding evidence
4	TF binding +DNase peak
5	TF binding or DNase peak
6	Motif hit

Note: This table presents the RegulomeDB variant classification scheme, which categorizes genomic variants based on their likelihood of affecting transcription factor (TF) binding and gene expression. Variants are classified into six main categories, ranging from those most likely to impact regulatory function (Category 1) to those with minimal binding evidence (Category 6).

- Category 1 variants are most likely to affect gene expression, incorporating evidence from expression quantitative trait loci (eQTLs), TF binding, DNase hypersensitivity, and motif matching.
- Category 2 variants are likely to affect TF binding but lack direct eQTL evidence.
- Category 3 variants have weaker evidence for disrupting binding, primarily based on TF motifs and DNase accessibility.
- Category 4–6 variants have progressively less supporting evidence, with Category 6 representing variants identified solely by motif presence.

Each classification integrates multiple lines of regulatory evidence, including:

- eQTL: Variants associated with gene expression changes.
- TF binding: Evidence from chromatin immunoprecipitation (ChIP) or other assays.
- Motif matching: Presence of a known TF binding sequence.
- DNase footprint/peak: Indications of open chromatin and potential regulatory activity.

Table S4: This table reports the allele frequency of seven nsSNPs in the coding region of PDCD1 across genetic ancestry groups and sexes.

- Genetic Ancestry Group: These groups represent individuals from diverse ancestral backgrounds, including European (Finnish and non-Finnish), African/African American, Admixed American, Ashkenazi Jewish, East Asian, Middle Eastern, Amish, South Asian, and other unspecified populations.
- Allele Count: Observed occurrences of the allele.
- Allele Number: Total alleles analyzed per group.
- Number of Homozygotes: Individuals with two copies of the allele (none observed).
- Allele Frequency: Proportion of the allele in each group (Allele Count / Allele Number).

1. rs1192883440/ L25V

Genetic Ancestry Group	Allele Count	Allele Number	Number of Homozygotes	Allele Frequency
European (Finnish)	1	61940	0	0.00001614
African/African American	0	74550	0	0.000
Admixed American	0	56402	0	0.000
Ashkenazi Jewish	0	29040	0	0.000
East Asian	0	43830	0	0.000
Middle Eastern	0	6014	0	0.000
European (non-Finnish)	0	1168886	0	0.000
Amish	0	910	0	0.000
South Asian	0	86918	0	0.000
Remaining	0	61630	0	0.000
XX	0	802710	0	0.000
XY	1	787410	0	0.000001270
**Total**	**1**	**1590120**	**0**	**6.289e-7**

Note:The allele was detected in European (Finnish) (1/61,940, frequency = 0.00001614) and XY individuals (1/787,410, frequency = 0.000001270), but absent elsewhere. The total allele frequency across all groups is 6.289×10^{-7}, indicating extreme rarity.

2.rs1700935524/ W67C

Genetic Ancestry Group	Allele Count	Allele Number	Number of Homozygotes	Allele Frequency
African/African American	1	74902	0	0.00001335
Admixed American	0	59980	0	0.000
Ashkenazi Jewish	0	29600	0	0.000
East Asian	0	44878	0	0.000
European (Finnish)	0	57572	0	0.000
Middle Eastern	0	6084	0	0.000
European (non-Finnish)	0	1179950	0	0.000
Amish	0	912	0	0.000
South Asian	0	91074	0	0.000
Remaining	0	62434	0	0.000
XX	1	808762	0	0.000001236
XY	0	798624	0	0.000
**Total**	**1**	**1607386**	**0**	**6.221e-7**

Note: The allele was detected in African/African American individuals (1/74,902, frequency = 0.00001335) and XX individuals (1/808,762, frequency = 0.000001236). The allele was absent in all other groups. The total allele frequency across all populations is 6.221×10^{-7}, indicating extreme rarity.

3. rs987449655/ L79V

Genetic Ancestry Group	Allele Count	Allele Number	Number of Homozygotes	Allele Frequency
European (non-Finnish)	2	1180002	0	0.000001695
African/African American	0	75068	0	0.000
Admixed American	0	60020	0	0.000
Ashkenazi Jewish	0	29604	0	0.000
East Asian	0	44886	0	0.000
European (Finnish)	0	62328	0	0.000
Middle Eastern	0	6062	0	0.000
Amish	0	912	0	0.000
South Asian	0	91082	0	0.000
Remaining	0	62478	0	0.000
XX	0	811462	0	0.000
XY	2	800980	0	0.000002497
**Total**	**2**	**1612442**	**0**	**0.000001240**

Note: The allele was detected in European (non-Finnish) individuals (2/1,180,002, frequency = 0.000001695) and XY individuals (2/800,980, frequency = 0.000002497). The allele was absent in all other groups. The overall allele frequency across all populations is 0.000001240, highlighting its rarity.

4. rs2124856605/ Y223S

Genetic Ancestry Group	Allele Count	Allele Number	Number of Homozygotes	Allele Frequency
Admixed American	1	60000	0	0.00001667
European (non-Finnish)	3	1179826	0	0.000002543
African/African American	0	75028	0	0.000
Ashkenazi Jewish	0	29574	0	0.000
East Asian	0	44872	0	0.000
European (Finnish)	0	63840	0	0.000
Middle Eastern	0	6054	0	0.000
Amish	0	912	0	0.000
South Asian	0	91066	0	0.000
Remaining	0	62476	0	0.000
XX	3	812168	0	0.000003694
XY	1	801480	0	0.000001248
**Total**	**4**	**1613648**	**0**	**0.000002479**

Note: The allele was detected in:Admixed American (1/6,000, frequency = 0.00001667), European (non-Finnish) (3/117,982, frequency = 0.00002543), XX individuals (3/812,168, frequency = 0.00003694) and XY individuals (1/801,480, frequency = 0.00001248).The allele was absent in African/African American, Ashkenazi Jewish, East Asian, European (Finnish), Middle Eastern, Amish, South Asian, and Remaining groups.The overall allele frequency across all populations is 0.000002479, indicating its rarity.

5. rs2124856279/ C241W

Genetic Ancestry Group	Allele Count	Allele Number	Number of Homozygotes	Allele Frequency
European (non-Finnish)	1	1179990	0	8.475e-7
African/African American	0	75040	0	0.000
Admixed American	0	60016	0	0.000
Ashkenazi Jewish	0	29598	0	0.000
East Asian	0	44880	0	0.000
European (Finnish)	0	63700	0	0.000
Middle Eastern	0	6062	0	0.000
Amish	0	912	0	0.000
South Asian	0	91088	0	0.000
Remaining	0	62504	0	0.000
XX	1	812258	0	0.000001231
XY	0	801532	0	0.000
**Total**	**1**	**1613790**	**0**	**6.197e-7**

Note: The allele was detected in: European (non-Finnish) (1/1,179,990, frequency = 8.475×10^{-7}) and XX individuals (1/812,258, frequency = 0.000001231). The allele was absent in all other genetic ancestry groups, including African/African American, Admixed American, Ashkenazi Jewish, East Asian, European (Finnish), Middle Eastern, Amish, South Asian, and Remaining. The total allele frequency across all populations is 6.197×10^{-7}, indicating its extreme rarity.

6. rs2124856153/I251T

Genetic Ancestry Group	Allele Count	Allele Number	Number of Homozygotes	Allele Frequency
European (non-Finnish)	1	1179912	0	8.475e-7
African/African American	0	74986	0	0.000
Admixed American	0	60016	0	0.000
Ashkenazi Jewish	0	29600	0	0.000
East Asian	0	44850	0	0.000
European (Finnish)	0	63538	0	0.000
Middle Eastern	0	6062	0	0.000
Amish	0	910	0	0.000
South Asian	0	91072	0	0.000
Remaining	0	62500	0	0.000
XX	1	812084	0	0.000001231
XY	0	801362	0	0.000
**Total**	**1**	**1613446**	**0**	**6.198e-7**

Note: The allele was detected in: European (non-Finnish) (1/1,179,912, frequency = 8.475×10^{-7}) XX individuals (1/812,084, frequency = 0.000001231). The allele was absent in all other genetic ancestry groups, including African/African American, Admixed American, Ashkenazi Jewish, East Asian, European (Finnish), Middle Eastern, Amish, South Asian, and Remaining. The total allele frequency across all populations is 6.198×10^{-7}, indicating its extreme rarity.

7. rs775100301/W286G

Genetic Ancestry Group	Allele Count	Allele Number	Number of Homozygotes	Allele Frequency
African/African American	2	74890	0	0.00002671
Admixed American	0	59910	0	0.000
Ashkenazi Jewish	0	29520	0	0.000
East Asian	0	44874	0	0.000
European (Finnish)	0	63176	0	0.000
Middle Eastern	0	5982	0	0.000
European (non-Finnish)	0	1179050	0	0.000
Amish	0	912	0	0.000
South Asian	0	90864	0	0.000
Remaining	0	62394	0	0.000
XX	1	811432	0	0.000001232
XY	1	800140	0	0.000001250
**Total**	**2**	**1611572**	**0**	**0.000001241**

Note: The allele was detected in: African/African American (2/74,890, frequency = 0.00002671), and XY individuals (1/800,140, frequency = 0.000001250) . The allele was absent in all other genetic ancestry groups, including Admixed American, Ashkenazi Jewish, East Asian, European (Finnish and non-Finnish), Middle Eastern, Amish, South Asian, and Remaining. The total allele frequency across all populations is 0.000001241, indicating its rarity.

Table S5: Allele frequency of SNPs in the 3' untranslated region (3'UTR) of the PDCD1 gene across different genetic ancestry groups and sexes.

1. rs543306494

Genetic Ancestry Group	Allele Count	Allele Number	Number of Homozygotes	Allele Frequency
Admixed American	1	17858	0	0.00005600
African/African American	2	45456	0	0.00004400
Ashkenazi Jewish	0	8580	0	0.000
East Asian	0	16576	0	0.000
European (Finnish)	0	10716	0	0.000
Middle Eastern	0	786	0	0.000
European (non-Finnish)	0	118172	0	0.000
Amish	0	912	0	0.000
South Asian	0	5548	0	0.000
Remaining	0	8870	0	0.000
XX	2	121668	0	0.00001644
XY	1	111806	0	0.000008944
**Total**	**3**	**233474**	**0**	**0.00001285**

Note:The allele was detected in: Admixed American (1/17,858, frequency = 0.00005600), African/African American (2/45,456, frequency = 0.00004400), XX individuals (2/121,668, frequency = 0.00001644) and XY individuals (1/111,806, frequency = 0.000008944). The allele was absent in all other ancestry groups. The overall allele frequency across all populations is 0.00001285, indicating the allele is rare in the studied population.

2. rs560497981

Genetic Ancestry Group	Allele Count	Allele Number	Number of Homozygotes	Allele Frequency
African/African American	102	45398	0	0.002247
Middle Eastern	1	782	0	0.001279
Admixed American	17	17764	0	0.0009570
Remaining	5	8822	0	0.0005668
European (non-Finnish)	1	117968	0	0.000008477
Ashkenazi Jewish	0	8508	0	0.000
East Asian	0	16514	0	0.000
European (Finnish)	0	10780	0	0.000
Amish	0	912	0	0.000
South Asian	0	5528	0	0.000
XX	60	121362	0	0.0004944
XY	66	111614	0	0.0005913
**Total**	**126**	**232976**	**0**	**0.0005408**

Note:The allele was most prevalent in: African/African American (102/45,398, frequency = 0.002247), Middle Eastern (1/782, frequency = 0.001279), Admixed American (17/17,764, frequency = 0.0009570), Remaining (5/8,822, frequency = 0.0005668) and European (non-Finnish) (1/117,968, frequency = 0.000008477). The allele was absent in Ashkenazi Jewish, East Asian, European (Finnish), Amish, and South Asian groups.Among XX individuals, the frequency was 0.0004944 (60/121,362), while among XY individuals, it was 0.0005913 (66/111,614). The total allele frequency across all populations is 0.0005408, suggesting that while rare overall, the allele is more prevalent in specific populations, particularly African/African American individuals.

3. rs550396273

Genetic Ancestry Group	Allele Count	Allele Number	Number of Homozygotes	Allele Frequency
African/African American	1	45618	0	0.00002192
Admixed American	0	18516	0	0.000
Ashkenazi Jewish	0	8656	0	0.000
East Asian	0	16684	0	0.000
European (Finnish)	0	10786	0	0.000
Middle Eastern	0	800	0	0.000
European (non-Finnish)	0	121290	0	0.000
Amish	0	912	0	0.000
South Asian	0	5846	0	0.000
Remaining	0	9154	0	0.000
XX	0	124054	0	0.000
XY	1	114208	0	0.000008756
**Total**	**1**	**238262**	**0**	**0.000004197**

Note: The allele was detected in: African/African American (1/45,618, frequency = 0.00002192) and XY individuals (1/114,208, frequency = 0.000008756). The allele was absent in all other ancestry groups. The total allele frequency across all populations is 0.000004197, indicating extreme rarity.
